# Genome mining for drug discovery: cyclic lipopeptides related to daptomycin

**DOI:** 10.1093/jimb/kuab020

**Published:** 2021-03-19

**Authors:** Richard H Baltz

**Affiliations:** CognoGen Biotechnology Consulting, 7757 Uliva Way, Sarasota, FL 34238, USA

**Keywords:** A54145, Actinomycete, *Actinoplanes*, Amphomycin, CDA, Combinatorial biosynthesis, Daptomycin, Friulimicin, Genome mining, Glycinocin, Malacidin, Parvuline, *Saccharomonospora*, *Streptomyces*, Taromycin, Telomycin

## Abstract

The cyclic lipopeptide antibiotics structurally related to daptomycin were first reported in the 1950s. Several have common lipopeptide initiation, elongation, and termination mechanisms. Initiation requires the use of a fatty acyl-AMP ligase (FAAL), a free-standing acyl carrier protein (ACP), and a specialized condensation (C^III^) domain on the first NRPS elongation module to couple the long chain fatty acid to the first amino acid. Termination is carried out by a dimodular NRPS that contains a terminal thioesterase (Te) domain (CAT-CATTe). Lipopeptide BGCs also encode ABC transporters, apparently for export and resistance. The use of this mechanism of initiation, elongation, and termination, coupled with molecular target-agnostic resistance, has provided a unique basis for robust natural and experimental combinatorial biosynthesis to generate a large variety of structurally related compounds, some with altered or different antibacterial mechanisms of action. The FAAL, ACP, and dimodular NRPS genes were used as molecular beacons to identify phylogenetically related BGCs by BLASTp analysis of finished and draft genome sequences. These and other molecular beacons have identified: (i) known, but previously unsequenced lipopeptide BGCs in draft genomes; (ii) a new daptomycin family BGC in a draft genome of *Streptomyces sedi*; and (iii) novel lipopeptide BGCs in the finished genome of *Streptomyces ambofaciens* and the draft genome of *Streptomyces zhaozhouensis*.

**Abbreviations**
A: adenylation domain
AA: amino acid
ACAD: acyl-CoA dehydrogenase superfamily
ACP: acyl carrier protein
ACP-MP: ACP multiprobe
BGC: biosynthetic gene cluster
C: condensation domain
CAT: NRPS elongation module
CATE: NRPS elongation module
CATTe: NRPS elongation/termination module
CDA: calcium-dependent antibiotic
clKyn: 4-Cl-kynurenine
cLP: cyclic lipopeptide
clTrp: 6-Cl-tryptophan
FA: fatty acid
FAAL: fatty acyl-AMP ligase
Hpg: hydroxy-phenylglycine
Hyd: hydrophobic amino acid
Kyn: kynurenine
LP: lipopeptide
M: methyltransferase domain
mAsp: methyl-aspartic acid
MbtH-MP: MbtH multiprobe
mGlu: methyl-glutamic acid
MOA: mechanism of action
moAsp: methoxy-aspartic acid
MRSA: methicillin-resistant *Staphylococcus aureus*
NP: natural product
NRP: nonribosomal peptide
NRPS: nonribosomal peptide synthetase
PCP: peptidyl carrier protein
PK: polyketide
PKS-I: type I polyketide synthetase
PPTase: phosphopantetheinyl transferase
Sar: sarcosine (*N*-methyl-glycine)
SAR: structure–activity relationship
SM: secondary metabolite
T: thiolation (PCP) domain
Te: thioesterase domain
TTe-MP: TTe multiprobe
UncBac: uncultured bacterium

## Introduction

For robust drug discovery and development, it has been historically productive to test derivatives or structural variants of compounds already proven to be clinically efficacious with low toxicity. This approach is well documented for natural products (NPs) (Katz & Baltz, 2016; Butler & Paterson, 2020; Newman & Cragg, 2020). For NPs approved for human medicine, animal health, or plant crop protection, over 60% are biosynthesized by type I polyketide synthase (PKS-I), nonribosomal peptide synthetase (NRPS), or mixed NRPS/PKS-I mechanisms (Katz & Baltz, 2016; Baltz, 2017c, 2019). Genome mining has provided a new paradigm for discovery of natural variants of known NPs, as well as novel NP biosynthetic gene clusters (BGCs) not expressed in standard fermentations (Challis, 2008, 2014; Baltz, 2008a, 2017c, 2019; Corre & Challis, 2009; Ikeda et al., 2014; Aigle et al., 2014; Bachmann et al., 2014; Doroghazi et al., 2014; Iftime et al., 2016; Ziemert et al., 2016). Genome mining has also provided a wealth of new PKS-I and NRPS parts and devices that can be exploited in combinatorial biosynthesis (Baltz, 2018; Yuzawa et al., 2018; Kudo et al., 2019; McErlean et al., 2019; Hwang et al., 2020). Both *de novo* discovery and combinatorial biosynthesis can be coupled with medicinal chemistry to further explore structure–activity relationships (SAR) for drug discovery and development. An example of an NRPS-derived commercial product that has been developed, and further modified by these approaches is the cyclic lipopeptide antibiotic daptomycin (Debono et al., 1988; Baltz, 2014b, 2014c, 2014d; Knight-Connoni et al., 2016).

Cyclic lipopeptide antibiotics produced by actinomycetes were first discovered in the 1950s (Baltz et al., 2005), and daptomycin was the first to be approved for treatment of Gram-positive infections, including methicillin-resistant *Staphylococcus aureus* (MRSA) (Baltz, 2009; Eisenstein et al., 2010). By the mid-2000s, NRPS BGCs encoding daptomycin (Dpt), A54145 (Lpt), and calcium-dependent antibiotic (CDA) had been cloned and sequenced (Hojati et al., 2002; Miao et al., 2005; Miao, Brost, et al., 2006). These lipopeptides have 10-membered ring structures with identical chirality (Hojati et al., 2002; Miao et al., 2005; Miao, Brost, et al., 2006; Gu et al., 2011), and all three NRPS multienzymes utilize phylogenetically related dimodular NRPS termination proteins (CAT-CATTe) that insert the final two amino acids, 3mGlu-Kyn (DptD), 3mGlu-Ile (LptD), or 3mGlu-Trp (CDA-PSIII), then cyclize and release the final products by thioesterase (Te) domains. Early combinatorial biosynthesis studies at Cubist Pharmaceuticals demonstrated that a *dptD* deletion mutant of *Streptomyces roseosporus* could be complemented by the *lptD* and CDA-*PSIII* genes from *Streptomyces fradiae* and *Streptomyces coelicolor*, respectively, to produce daptomycin analogs containing Ile or Trp in the terminal amino acid position (Miao, Coëffet-Le Gal, et al., 2006). These findings were followed by a series of studies on combinatorial biosynthesis in *S. roseosporus* (Nguyen, Kau, et al., 2006; Nguyen, Ritz, et al., 2006; Coëffet-Le Gal et al., 2006; Doekel et al., 2008) and *S. fradiae* (Nguyen et al., 2010; Alexander et al., 2010, 2011) that generated many active lipopeptide antibiotics related to daptomycin and A54145, including several with highly improved efficacy in a *Streptococcus pneumoniae* murine lung infection model, while maintaining the high antibacterial activity against multiple Gram-positive pathogens and low toxicity of daptomycin (Baltz, 2014b, 2014c).

The NRPS genes (*dptA, dptBC*, and *dptD*) in the daptomycin BGC are preceded by *dptE* and *dptF*, which encode a fatty acyl-AMP ligase (FAAL) and a free-standing acyl carrier protein (ACP) involved in initiation of lipopeptide assembly by coupling long chain fatty acids to the *N*-terminal Trp (Miao et al., 2005; Wittmann et al., 2008; Baltz, 2014b). Initiation of A54145 biosynthesis is carried out in a similar manner by an apparently fused FAAL-ACP encoded by *lptEF* (Miao, Brost, et al., 2006). However, recent genome mining studies indicate that the fragmented A54145 BGCs in draft genome assemblies of four other *Streptomyces* species encode free-standing FAALs and ACPs (Baltz, 2018; This report). CDA biosynthesis does not use a FAAL-ACP mechanism for initiation of lipopeptide assembly (Hojati et al., 2002).

Additional cyclic lipopeptide BGCs have been sequenced and annotated more recently, and several employ initiation and termination mechanisms similar to those utilized for daptomycin and A54145 assembly (Müller et al., 2007; Wang et al., 2011; Yamanaka et al., 2014; Fu et al., 2015; Johnston et al., 2016; Liu et al., 2016; Hover et al., 2018; Reynolds et al., 2018). This report explores the evolutionary relationships between these structurally diverse but evolutionarily related cyclic lipopeptides (Baltz, 2008b), particularly as it relates to biosynthetic features that can be exploited by recent advancements in synthetic biology, genome mining, and combinatorial biosynthesis for drug discovery (Baltz, 2018; Katz et al., 2018).

## Materials and Methods

 

### Strains and Lipopeptide BGC Sequencing Status

The DNA sequencing status of select actinomycete strains and uncultured bacteria, and their lipopeptide BGCs are summarized in Table 1.

**Table 1:** Genome and Lipopeptide BGC Sequence Status for Actinomycetes and Uncultured Bacteria

Microorganism	Lipopeptide (predicted)	BGC status^a^	Genome status	Reference
*Streptomyces roseosporus* NRRL 11379	Daptomycin	Finished	Draft	Baltz (2017), Miao et al. (2005), Penn et al. (2006)
*Saccharomonospora* sp. CNQ490	Taromycin	Finished	Draft	Reynolds et al. (2018), Yamanaka et al. (2014)
*Saccharomonospora viridis* DSM 43017	(desCl-Taromycin)	Finished	Finished	Baltz (2010, 2018), Pati et al. (2009)
*Streptomyces fradiae* A54145	A54145	Finished	ND^b^	Miao, Brost, et al. (2006)
*Streptomyces exfoliatus* SM41693	(A54145)	Fragmented	Draft	Baltz (2018)
*Streptomyces griseoluteus* ISP-5360	(A54145)	Fragmented	Draft	Baltz (2018)
*Streptomyces pini* PL19	(A54145)	Fragmented	Draft	Baltz (2018)
*Streptomyces barkulensis* RC 1830	(A54145)	Fragmented	Draft	This report
*Streptomyces coelicolor* A3(2)	CDA	Finished	Finished	Hojati et al. (2002)
*Streptomyces lividans* TK24	CDA	Finished	Finished	Ho et al. (2002), Penn et al. (2006), Rückert et al. (2015)
*Streptomyces* sp. MBT28	(CDA)	Fragmented	Draft	Baltz (2018)
*Streptomyces* sp. NRRL WC-3795	(CDA)	Fragmented	Draft	Baltz (2018)
*Actinoplanes friuliensis* DSM 7358	Friulimicin	Finished	Finished	Müller et al. (2007)
*Uncultured bacterium* GQ475284	Friulimicin	Finished	NA^c^	Kim et al. (2010)
*Streptomyces viridochromogenes* ATCC 29814	Laspartomycin^d^	Finished	Draft	Wang et al. (2011)
*Streptomyces* sp. M56	(Glycinocin)^d^	Draft (AS)^e^	Finished	Kim et al. (2014), This report
*Streptomyces malaysiensis* DSM 4137	(Glycinocin)	Draft (AS)	Finished	This report
*Streptomyces* sp. 1331.2	(Glycinocin)	Fragmented	Draft	This report
*Streptomyces* sp. SPMA113	(Glycinocin)	Draft (AS)	Draft	Komaki et al. (2016), This report
*Uncultured bacterium* KY654519	Malacidin	Finished	NA	Hover et al. (2018)
*Uncultured bacterium* KF264539	(Malacidin)	Finished?	NA	Owen et al. (2013)
*Streptomyces canus* ATCC 12646	Telomycin	Finished	Draft	Fu et al. (2015)
*Streptomyces canus* ATCC 12647	Telomycin	Finished	Draft	Johnstone et al. (2016), Liu et al. (2016)
*Streptomyces qaidamensis* S10	(Telomycin)	Fragmented	Draft	Zhang et al. (2018)
*Streptomyces formicae* KY5	(Telomycin)	Draft (AS)	Finished	Holmes et al. (2018), This report
*Streptomycces fungicidicus* ATCC 21013	Enduracidin	Finished	ND	Yin & Zabriski (2006)
*Streptomyces canus* ATCC 12237^f^	Amphomycin^g^	Fragmented	Draft	Baltz et al. (2005), This report
*Streptomyces parvulus* 2297	(Parvuline)^g^	Fragmented	Draft	Baltz et al. (2005), Hu et al. (2018), This report
*Streptomyces ambofaciens* ATCC 23877	(Lipotridecapeptide)	Draft (AS)	Finished	Aigle et al. (2014), Thibessard et al. (2015), This report
*Streptomyces zhaozhouensis* CGMCC 4.7095	(Unknown)	Fragmented	Draft	He et al. (2014), This report
*Streptomyces sedi* JCM 16909	(Unknown)	Fragmented	Draft	Li et al. (2009), This report

^a^NCBI Genome (https://www.ncbi.nlm.nih.gov/genome/).

^b^ND, not done.

^c^NA, not assembled.

^d^Laspartomycin (Las) is a member of the glycinocin (Gly) family of lipopeptides that share a common peptide (Baltz et al., 2005).

^e^AS, draft BGC from antiSMASH 5.0 (Blin et al., 2019).

^f^Same as DSM 40017.

^g^Parvuline is a member of the amphomycin family of lipopeptides that also includes A-1437, tsushimycin, and aspartocin (Baltz et al., 2005). The amphomycin family differs from friulimicins at amino acid position 1 (Asp_1_ in amphomycin, Asn_1_ in friiulimicin).

### BLASTp analysis

Amino acid sequence similarities were determined by BLASTp analyses (Altschul et al., 1990) on the National Center for Bioinformatic Information (NCBI) web server (http://blast.ncbi.nim.nih.gov/Blast.cgi).

### Searches for Cryptic Lipopeptide BGCs

Initial BLASTp searches of genome sequences in NCBI were carried out using various molecular beacons (Supplementary Table S1), including genes from the daptomycin BGC, and homologs from other lipopeptide producers. Putative lipopeptide producers were also surveyed for the presence of MbtH homologs related to those of known lipopeptide producers by BLASTp analysis with a 24-mer MbtH multiprobe (Baltz, 2014a, 2017a). Other BLASTp searches were carried out with pathway-specific genes from other lipopeptide BGCs to help distinguish between known and novel lipopeptide BGCs. Putative lipopeptide BGCs were analysed from finished genomes by antiSMASH 4.0 or 5.0 (Blin et al., 2017, 2019).

### ACP Multiprobe Analysis

An ACP multiprobe was prepared by concatenating free-standing ACP amino acid sequences from 19 BGCs encoding 7 different lipopeptides as follows: daptomycin, position 1; taromycin, 2–3; A54145, 4–8; friulimicin, 9–10; laspartomycin/glycinocin, 11–13; malacidin, 14–15; and telomycin, 16–19. The numbers of ACPs for each lipopeptide reflect the sequences from BGCs previously published or from BGCs identified in this report. The lipopeptide producing actinomycetes and uncultured bacteria are listed in Table 1, and the multiprobe sequence in Supplementary Table S2. The multiprobe was used for BLASTp analysis of lipopeptide producers, and the 19 individual color readouts of pink, green, blue, and black, were converted to 4, 3, 2, and 1 to prepare numerical codes.

### NRPS Amino Acid Binding Pocket Analysis

Analysis of NRPS adenylation (A) domain amino acid binding pocket specifities (Stachelhaus et al., 1999; Challis et al., 2000) was carried out using NRPSpredictor2 (Rottig et al., 2011).

## Results

 

### Cyclic Lipopeptide BGCs for Comparative Analysis

**Fig. 1:**
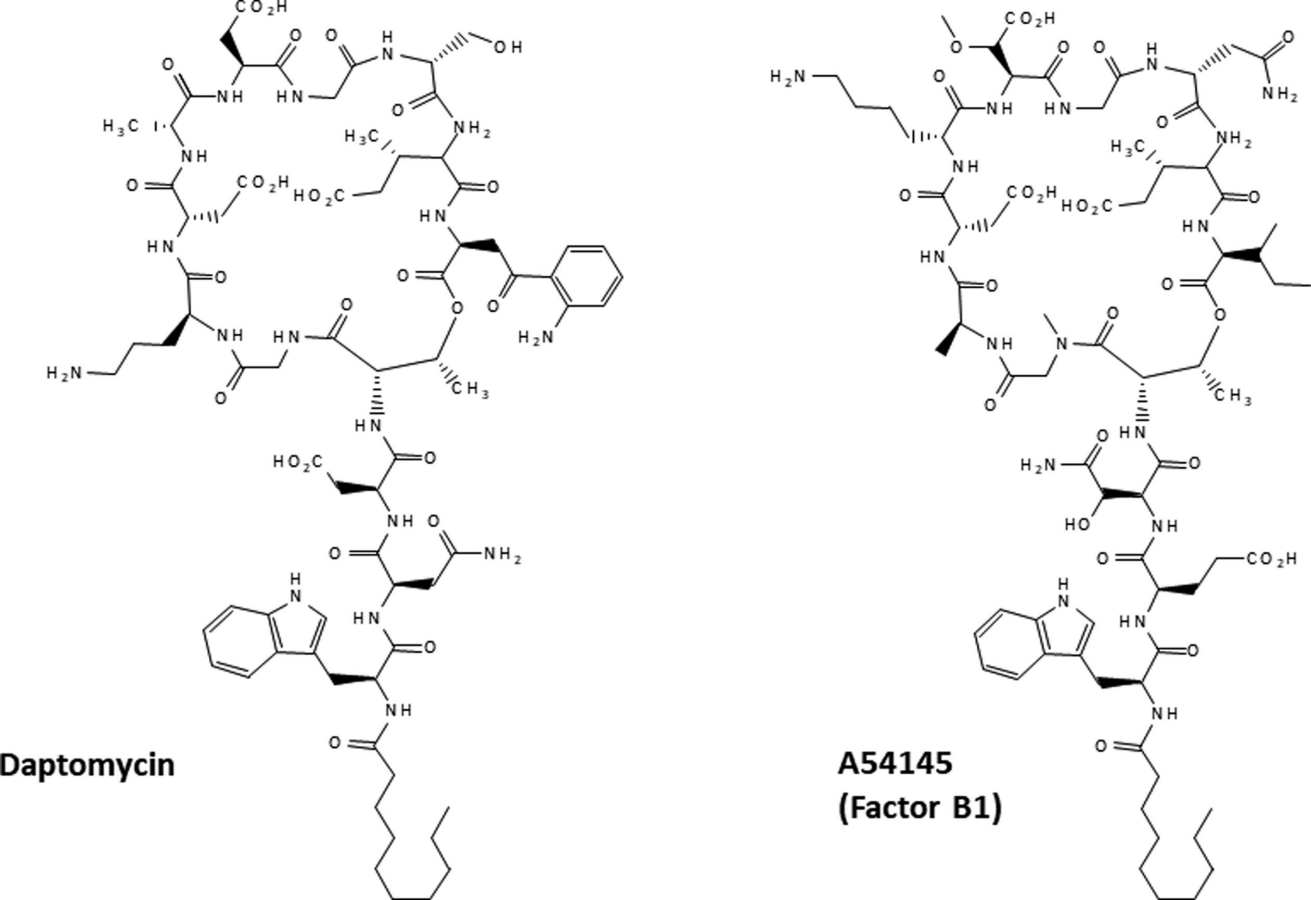
Structures of daptomycin and A54145 B1.

**Fig. 2:**
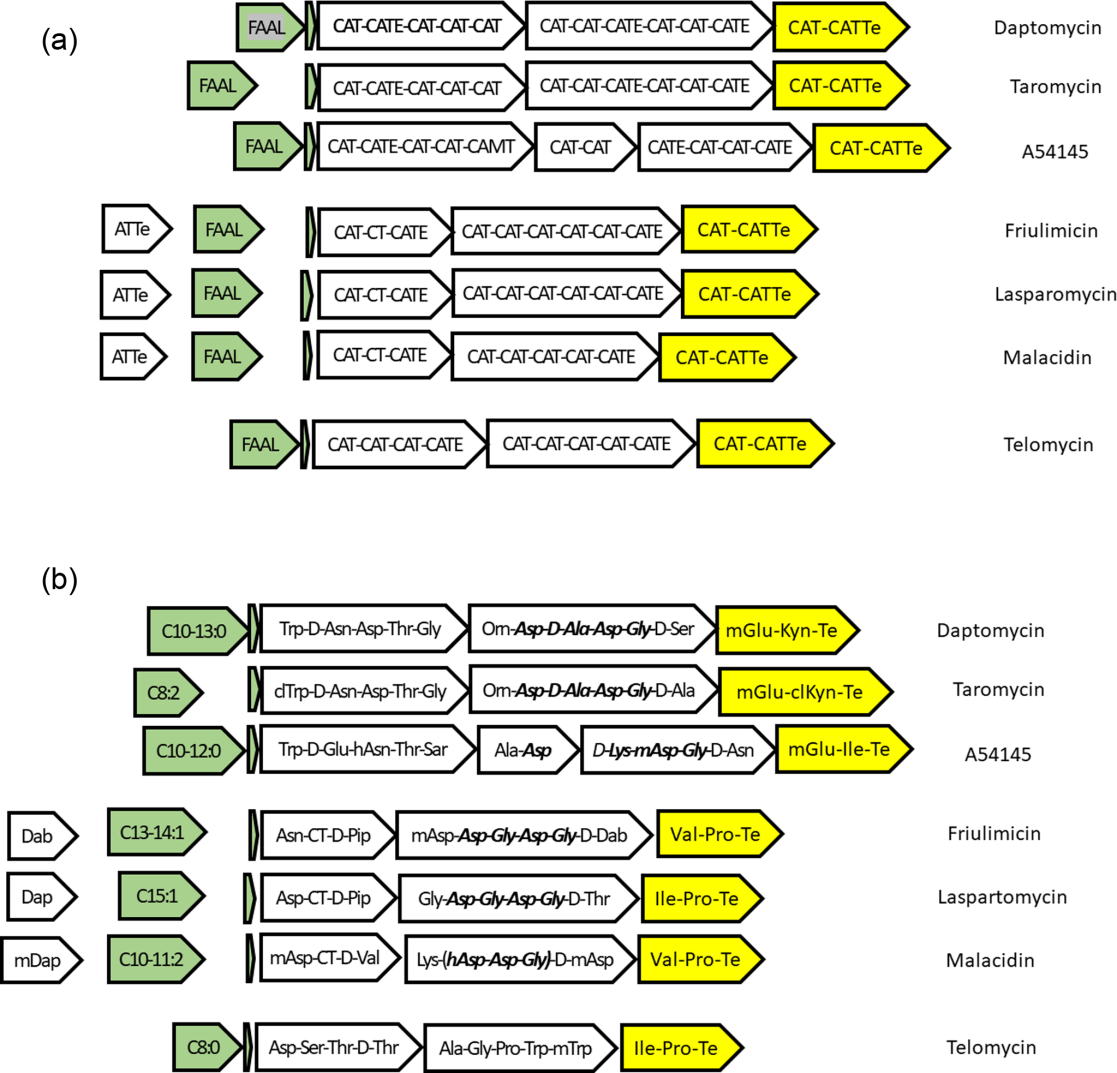
Organization of genes encoding lipopeptide initiation, elongation, and termination in actinomycetes. (a) Locations of genes encoding FAAL, ACP (small arrow), and NRPS subunits showing module and domain structures. ACAD family fatty acid dehydrogenases are located in the gaps between FAAL and ACP genes for taromycin, friulimicin, laspartomycin, and malacidin. Trans ATTe tri-domains interact with CT modules at position 2 for friulimicin, laspartomycin, and malacidin. (b) Fatty acid and amino acid specificities of FAAL enzymes and NRPS modules. Note that friulimicin and laspartomycin have a single double bond, whereas taromycin and malacidin have two double bonds in long chain length fatty acids.

The structures of daptomycin and A54145 are shown as examples of cyclic lipopeptide antibiotics in Fig. 1. For comparative analysis, key elements of lipopeptide assembly machines for daptomycin, taromycin, A54145, friulimicin, laspartomycin/glycinocin, malacidin, and telomycin are shown in Fig. 2a and b. The key elements include molecular parts and devices for initiation, elongation, and termination/release of the finished lipopeptides. Other conserved accessory devices include ABC transporters for export and resistance, and MbtH-like chaperones. The status of assembly of the genomes and BGCs encoding these lipopeptides is presented in Table 1, and background information on each molecule is provided below.

#### Daptomycin

Daptomycin is a Ca^2+^-dependent cyclic lipopeptide antibiotic produced commercially by *S. roseosporus*. It has been approved to treat skin and skin structure infections, bacteremia, and right-sided endocarditis caused by Gram-positive pathogens, including MRSA (Baltz, 2009; Eisenstein et al., 2010). Daptomycin has a 10-membered peptidic ring, which includes the tetrapeptide Asp-d-Ala-Asp-Gly for Ca^2+^-binding (Fig. 2b), and a three amino acid exocyclic tail attached *N*-terminally to decanoic acid (Fig. 1). *S. roseosporus* has a preference for incorporation of C11–C13 branched chain saturated fatty acids in the absence of decanoic acid feeding (Baltz et al., 2005). *S. roseosporus* has been developed into a designer host chassis for facile lipopeptide combinatorial biosynthesis (Nguyen, Ritz, et al., 2006; Coëffet-Le Gal et al., 2006; Doekel et al., 2008; Baltz, 2014b).

#### Taromycin

Taromycin is a cyclic lipopeptide antibiotic produced by the marine *Saccharomonospora* sp. CNQ490 (Yamanaka et al., 2014; Baltz, 2018; Reynolds et al., 2018). Taromycin is closely related to daptomycin, and differs in the tridecapeptide by a single amino acid substitution (d-ala_11_ for d-ser_11_), by chlorination of l-Trp_1_ and l-Kyn_13_, and it has a C8 fatty acid side chain unsaturated in two positions (Reynolds et al., 2018; Yamanaka et al., 2014). Its Ca^2+^ binding tetrapeptide is identical to that of daptomycin (Fig. 2b). A cryptic taromycin-like BGC, but lacking the tryptophan chlorinase gene involved in chlorination of Trp and Kyn in taromycin, is encoded by *Saccharomonospora viridis* DSM 43017, a causative agent of Farmer's Lung Disease (Pati et al., 2009; Baltz, 2010b, 2018).

#### A54145

A54145 is a 10-membered Ca^2+^-dependent cyclic lipopeptide antibiotic distantly related to daptomycin (Boeck et al., 1990) that shares the same chirality as daptomycin and taromycin (Miao, Brost, et al., 2006; Gu et al., 2011; Yamanaka et al., 2014; Reynolds et al., 2018). It has a Ca^2+^-binding sequence of Asp-d-Lys-moAsp-Gly (Fig. 2b). The producing strain, *S. fradiae* A54145, has been developed into a designer host chassis for facile lipopeptide combinatorial biosynthesis (Alexander et al., 2010, 2011; Baltz, 2014b; Nguyen et al., 2010). Recent bioinformatic studies indicate that four other *Streptomyces* species encode the A54145 BGC, and could serve as sources of parts and devices for combinatorial biosynthesis (Baltz, 2018; This report).

#### Calcium-dependent antibiotic

CDA is a Ca^2+^-dependent cyclic lipopeptide antibiotic produced by *S. coelicolor* (Hojati et al., 2002) and *Streptomyces lividans* (Ho et al., 2002; Penn et al., 2006), and its BGC is encoded in other *Streptomyces* sp. (Baltz, 2018) (Table 1). It has a 10-membered peptidic ring with a single exocyclic amino acid attached to epoxy-hexanoic acid. CDA assembly is not initiated by the FAAL:ACP mechanism used by other lipopeptides (Hojati et al., 2002).

#### Laspartomycin

Laspartomycin is a 10-membered Ca^2+^-dependent cyclic lipopeptide antibiotic produced by *Streptomyces viridochromogenes* ATCC 29814. It was first described in the 1950s (Baltz et al., 2005), and its BGC has been sequenced (Wang et al., 2011). It is a member of the glycinocin family (Baltz et al., 2005). It has a single exocyclic amino acid coupled to a mono-unsaturated long chain fatty acid. The laspartomycin cyclic peptide backbone has the same chirality as daptomycin, taromycin, A54145, and CDA, and has a canonical Ca^2+^-binding tetrapeptide, Asp-Gly-Asp-Gly (Fig. 2b).

#### Friulimicin

Friulimicin is a 10-membered Ca^2+^-dependent cyclic lipopeptide antibiotic produced by *Actinoplanes friuliensis* DSM 7358. Friulimicin is structurally related to amphomycin and parvuline, differing at the exocyclic amino acid (Asn in friulimicin and Asp in amphomycin/parvuline), and sharing identical Asp-Gly-Asp-Gly Ca^2+^-binding tetrapeptides (Baltz et al., 2005). Its BGC was published in 2005 (Müller et al., 2007). The BGCs for amphomycin and parvuline have not been published and analysed, but see below.

#### Telomycin

Telomycin was first described in the 1950s by scientists at Bristol-Myers (Misiek et al., 1957–1958). It is a 9-membered cyclic depsipeptide with a two amino acid exocyclic tail lacking a lipid side chain. Recent studies indicate that telomycin biosynthesis initiates with the coupling of a long chain fatty acid to the first amino acid and that the lipid is removed after the cyclic lipopeptide is released from the NRPS multienzyme (Fu et al., 2015). Two strains of *Streptomyces canus* were deposited by Bristol–Myers to support patent applications, and the telomycin BGCs have been sequenced from both strains (Fu et al., 2015; Johnston et al., 2016; Liu et al., 2016). The telomycin BGCs were chosen for inclusion in this analysis because they encode homologs to DptE, DptF, and DptD for initiation and termination of assembly, but the final cyclic peptide has no apparent Ca^2+^-binding tetrapeptide (Fig. 2b).

#### Malacidin

Malacidin is a cyclic lipopeptide antibiotic recently discovered from an uncultured bacterium (Hover et al., 2018). Malacidin has an 8-membered amino acid heterocycle and a two amino acid exocyclic tail coupled to a di-unsaturated fatty acid. Its lipopeptide assembly apparatus, including the use of DptE, DptF, and DptD homologs, is similar to those of friulimicin and laspartomycin (Fig. 2a and b), but it lacks a canonical Ca^2+^-binding tetrapeptide. Nonetheless, it requires high levels of Ca^2+^ for antibacterial activity (Hover et al., 2018).

### Components for Cyclic Lipopeptide Assembly

From a synthetic biology perspective, cyclic lipopeptide antibiotic assembly requires a number of parts and devices to build the assembly machines in microbial host chassis. In addition, lipopeptide biosynthesis often requires the coordinated acquisition of accessory devices for lipid or amino acid modifications, activation of ACPs and peptidyl carrier proteins (PCPs or T domains) by phosphopantetheinyl transferases (PPTases), MbtH chaperone function, host resistance, and transport. Typical Ca^2+^-dependent cyclic lipopeptides require an additional tetrapeptide device within the peptide ring to bind Ca^2+^ ions. Therefore, key components for lipopeptide assembly include: (i) fatty acid to amino acid coupling devices; (ii) multiple types of amino acid to amino acid coupling devices; (iii) parts to set chirality; (iv) devices to impart Ca^2+^-binding; (v) devices to cyclize and release lipopeptides from the giant multi-modular, multi-subunit NRPS assembly machines; and (vi) multiple accessory devices to facilitate the process. As the individual lipopeptide assembly functions are modular, they lend themselves to combinatorial evolutionary processes that can be accelerated by many orders of magnitude in the laboratory by combinatorial biosynthesis (Baltz, 2014b). In the following sections, I discuss evolutionary relationships that can be deduced from the analysis of the BGCs from structurally diverse, but evolutionarily related lipopeptide antibiotics produced by actinomycetes or uncultured bacteria.

### Activation and Coupling of Fatty Acids to Amino Acids (Initiation)

At the front end of lipopeptide assembly is the attachment of a long chain-length fatty acid to the first amino acid to initiate assembly. The evolution of this process was undoubtedly a key element in the evolution of lipopeptide assembly machines. Bioinformatic analysis of the daptomycin BGC identified three NRPS genes, *dptA, dptBC*, and *dptD* (Miao et al., 2005). Just upstream of *dptA* are *dptE* and *dptF*, which were initially annotated as acyl-CoA ligase and free standing ACP, respectively (Miao et al., 2005). Subsequent biochemical studies (Wittmann et al., 2008) showed that DptE has two activities that do not involve acyl-CoA intermediates. DptE activates certain long chain fatty acids with ATP to form fatty acyl-AMP intermediates; the fatty acids are then transferred to a holo-ACP (DptF) for subsequent coupling to l-Trp_1_ by a specialized C^III^ condensation domain of the first module of DptA (Miao et al., 2005). So DptE has two activities, FAAL and acyl-ACP synthetase (AAS) (Wittmann et al., 2008). For simplicity I refer to this type of enzyme as FAAL as it is typically annotated in NCBI. Also, mechanistic studies showed that DptE requires DptF for FAAL activity (Wittmann et al., 2008). The *dptE* and *dptF* genes are transcribed along with *dptABCD* genes as a single long transcript from a promoter upstream of *dptE* (Coëffet-Le Gal et al., 2006). This FAAL mechanism to activate long chain fatty acids by DptE is similar to the FAAL mechanism (FadD32) involved in mycolic acid biosynthesis in *Mycobacterium tuberculosis* (Kuhn et al., 2016). It differs in that the activated long chain fatty acid is transferred to an ACP in a small PKS (ACP-KS-AT-Te) in *M. tuberculosis*. DptE and FadD32 show 34% sequence identity in BLASTp analysis, indicating that they are distantly related evolutionarily.

The FAAL:ACP:C^III^ mechanism to initiate lipopeptide assembly is observed in other cyclic lipopeptide BGCs (Fig. 2a), including A54145, taromycin, friulimicin, laspartomycin/glycinocin, malacidin, telomycin, and enduracidin (not shown). The FAAL:ACP:C^III^ mechanism utilizes free-standing holo-ACPs activated by PPTases (Wittmann et al., 2008). It differs mechanistically from holo-ACPs and holo-PCPs involved in PKS and NRPS chain elongation in that it interacts with upstream FAAL enzymes and downstream specialized C^III^ domains. As such, they might be more closely related phylogenetically to each other than to the more common ACPs and PCPs imbedded in PKS or NRPS enzymes. However, they process fatty acids of different chain length and degree of saturation, and couple the fatty acids to different amino acids, so we might expect divergent ACP amino acid sequence relationships based on substrate and coupling partner preferences. The same might hold true for FAAL enzymes that must select fatty acids of correct chain length and degree of unsaturation from primary metabolic FA pools to bind and activate.

#### FAALs

Table 2 shows the amino acid sequence similarities between FAAL enzymes involved in lipopeptide assembly. Table 2 includes compounds validated chemically and others from BGCs identified bioinformatically. The four apparent LptE orthologs from *Streptomyces exfoliatus, Streptomyces griseoluteus, Streptomyces pini*, and *Streptomyces barkulensis* showed 79–90% amino acid sequence identities to the LptE domain of LptEF from *S. fradiae*, which otherwise showed only 46–52% sequence identities to FAAL sequences from other lipopeptide producers. The two LipA FAALs from friulimicin producers share 92% sequence identities, and LipA from *A. friuliensis* showed the highest sequence identities to paralogous FAALs encoded by strains harboring laspartomycin/glycinocin BGCs (59–62%). The four FAALs from strains identified bioinformatically to encode laspartomycin/glycinocin BGCs showed 73–78% sequence identities to LipA from the laspartomycin BGC in *S. viridochromogenes*, suggesting that they may be orthologs. The strains harboring telomycin BGCs encode FAALs sharing 75–99% sequence identities with Tem18 from *S. canus*, and are not closely related to any others. MlcG from the uncultured malacidin producer shows 95% sequence identity with a FAAL from a predicted malacidin BGC from another uncultured bacterium, but only 45–55% sequence identities with other FAALs. A particularly interesting pair of FAALs is those from the daptomycin and taromycin BGCs. Even though these BGCs encode very similar tridecapeptides, DptE shows only 46 sequence identity to Tar4. Curiously, DptE shows higher sequence identities to LptE orthologs encoded by five different A54145 BGCs (51–52%). These data likely reflect the large divergence in fatty acid preferences displayed by daptomycin and taromycin (Fig, 2B).

**Table 2:** DptE Homolog BLASTp Scores for Fatty Acyl-AMP Ligase (FAAL) Enzymes from Actinomycetes and Uncultured Bacteria

		Query protein^a^							
Actinomycete	FAAL (predicted protein)	DptE	Tar4	LptEF^b^	LipA(*Afr*i)	LipA (*Svir*)	LipA (M56)	Tem18	MlcG
*S. roseosporus* NRRL 11379	DptE	**100**	*46*	52	48	47	50	47	48
*Sa.* sp. CNQ490	Tar4	46	**100**	48	43	45	44	46	46
*Sa. viridis* DSM 43017	(Tar4)	47	**74**	47	41	46	46	48	45
*S. fradiae* A54145	LptEF	52	48	**100**	48	47	48	48	48
*S. exfoliatus* SM41693	(LptE)	51	49	**89**	48	50	49	49	49
*S. griseoluteus* ISP-5360	(LptE)	51	48	**90**	48	49	49	49	49
*S. pini* PL19	(LptE)	51	48	**79**	47	49	49	47	50
*S. barkulensis* RC 1830	(LptE)	52	49	**80**	48	48	49	47	50
*S. coelicolor* A3(2)	–	–	–	–	–	–	–	–	–
*S. lividans* TK24	–	–	–	–	–	–	–	–	–
*S.* sp. MBT28	–	–	–	–	–	–	–	–	–
*S.* sp. NRRL WC–3795	–	–	–	–	–	–	–	–	–
*A. friuliensis* DSM 7358	LipA-fri	48	43	48	**100**	59	61	53	52
*UncBac* GQ475284	(LipA-fri)	46	43	48	**92**	59	59	53	52
*S. viridochromogenes* ATCC 29814	(LipA-las)	47	45	47	59	**100**	**77**	54	54
*S. malaysiensis* DSM 4137	(LipA-gly)	50	45	49	61	**78**	**99**	54	54
*S.* sp. M56	(LipA-gly)	50	44	48	61	**77**	**100**	54	54
*S.* sp. SPMA113	(LipA-gly)	50	45	49	61	**78**	**99**	54	54
*S.* sp. 1331.2	(LipA-gly)	49	44	48	62	**73**	**75**	56	54
*S. canus* ATCC 12646	Tem18	47	46	48	53	54	54	**100**	55
*S. canus* ATCC 12647	Tlo18 = Tem18	47	46	48	53	54	54	**99**	55
*S. qaidamensis* S10	(Tem18)	47	46	48	53	54	55	**92**	53
*S. formicae* KY5	Tem18	46	45	46	52	53	54	**75**	53
*UncBac* KY654519	MlcG	48	46	48	52	54	54	55	**100**
*UncBac* KF264539	(MlcG)	48	45	48	53	53	54	55	**95**
*S. fungicidicus* ATCC 21013	ABD65965 (ORF45}^c^	42	44	42	41	49	39	40	39
*S. canus* ATTC 12237	KUN68831^b^	45	45	48	62	77	76	57	55
*S. parvulus* 2297	WP_114531151^b^	48	45	47	61	74	75	57	54
*S. ambofaciens* ATCC 23877	AKZ58686	49	46	50	53	56	57	57	56
*S. zhaozhouensis* CGMCC 4.7095	SOD64433	45	43	47	51	48	48	52	50
*S. sedi* JMC 16909	WP_139649592^a^	56	52	52	48	48	52	48	51

^a^Possible orthologs are shown in bold.

^b^These proteins share 82% sequence identities.

^c^Orf45 has a fused FAAL-ACAD (Yin & Zabriski, 2006).

#### Free-standing ACPs

ACPs and PCPs (T domains) are very important in the assembly of polyketide (PK) and nonribosomal peptide (NRP) secondary metabolites by PKS-I and NRPS multienzymes. In these cases, they are embedded in multimodular, multisubunit megaenzymes (Weissman, 2015; Marahiel, 2016; Süssmuth & Mainz, 2017; McErlean et al., 2019). The stand-alone ACPs involved in coupling fatty acids to amino acids in lipopeptide assembly present a striking contrast. Typical ACPs and PCPs have multiple protein-protein interactions in PK and NRP assembly, but differ in specificity from the stand alone ACPs involved in lipopeptide assembly. The latter interact with FAAL enzymes and specialized C^III^ domains (Miao et al., 2005; Miao, Brost, et al., 2006; Baltz, 2014b) involved in initiation of lipopeptide assembly. As such, they show little amino acid conservation with typical ACP and PCP domains in PKS-I and NRPS BGCs. This aspect of stand-alone ACPs, coupled with their small sizes (∼90 amino acids), makes them attractive molecular beacons to help identify known, related, and novel lipopeptide BGCs in finished and draft genomes. Table 3 shows the results of BLASTp analyses of different actinomycetes with DptF (ACP) homologs from seven lipopeptide BGCs that use both FAAL:ACP:C^III^ initiation and CAT-CATTe di-modular termination mechanisms. It is apparent that the ACP proteins are much more divergent than the FAAL enzymes (Table 2), and other proteins involved in lipopeptide assembly discussed below. This high level of amino acid sequence divergence has been exploited by generating an ACP multiprobe that can be used to help identify known, related, and novel lipopeptide BGCs in finished and draft genomes.

**Table 3:** DptF (ACP) Homolog BLASTp Scores in Actinomycetes and Uncultured Bacteria

		Query ACP protein^a^						
Microorganism	ACP (predicted)	DptF	Tar7	LptF^b^	LipD-fri	LipD-las	Tem19	MlcJ
*S. roseosporus* NRRL 11379	DptF	**100**	38	50	34	39	32	41
*S.* sp. CNQ490	Tar7	38	**100**	34	41	38	28	42
*S. viridis* DSM 43017	(Tar7)	38	**75**	34	41	39	27	45
*S. fradiae* A54145	LptEF	50	34	**100**	39	38	33	36
*S. exfoliates* SM41693	(LptF)	40	29	**84**	43	39	33	38
*S. griseoluteus* ISP-5360	(LptF)	39	30	**86**	46	42	28	39
*S. pini* PL19	(LptF)	39	30	**78**	42	39	28	41
*S. barkulensis* RC 1830	(LptF)	41	31	**81**	42	39	32	41
*A. friuliensis* DSM 7358	LipD-fri	34	41	39	**100**	70	32	50
*UncBac* GQ475284	(LipD-fri)	35	41	42	**93**	71	35	51
*S. viridochromogenes* ATCC 29814	LipD-las	39	38	38	70	**100**	40	47
*S. malaysiensis* DSM 4137	(LipD-las)	33	32	38	65	**80**	37	44
*S.* sp. M56	(LipD-las)	33	32	38	65	**80**	37	44
*S.* sp. SPMA113	(LipD-las)	33	32	38	65	**80**	37	44
*S.* sp. 1331.2	(LipD-las)	31	37	35	61	**80**	35	45
*S. canus* ATCC 12646	Tem19	32	28	29	33	41	**100**	36
*S. canus* ATCC 12647	Tlo19 = Tem19	32	31	29	33	39	**99**	36
*S. qaidamensis* S10	(Tem19)	33	28	27	34	39	**92**	36
*S. formicae* KY5	(Tem19?)	27	26	33	36	36	57	37
*UncBac* KY654519	MlcI	41	45	37	50	47	33	**100**
*UncBac* KF264539	MlcI	45	45	36	50	51	34	**88**
*S. fungicidicus*	ABD65965	37	42	37	47	44	43	39
*S. canus* ATCC 12237	KUN68828^c^	33	35	38	66	68	38	44
*S. parvulus* 2297	WP_114531148^c^	36	36	33	63	68	36	48
*S. ambofaciens* ATCC 23877	WP_053138555	34	36	30	49	55	37	49
*S. zhaozhouensis* CGMCC 4.7095	SOD64425	34	32	33	43	43	52	40
*S. sedi* JMC 16909	WP_139649588	50	47	51	34	37	35	48

Abbreviations: LipD-fri, ACP from friulimicin BGC; LipD-las, ACP from laspartomycin/glycinocin BGC.

^a^Possible orthologs are shown in bold.

^b^LptF, amino acids 651–732 of LptEF.

^c^These proteins share 78% sequence identities.

Table 4 shows results from ACP multiprobe analyses of the free-standing ACPs from the 19 lipopeptide BGCs that make up the multiprobe, and six others. The multiprobe is a contatenane including (sequentially) ACPs from the following BGCs: one from daptomycin, two from taromycins, five from A54145s, two from friulimicins, three from laspartomycin/glycinocins, two from malacidins, and four from telomycins. The degree of sequence similarity is reflected in the numerical code, from the highest (4) to the lowest (1) (see Materials and Methods). The first three ACP codes considered are those of highly related daptomycin, taromycin, and a taromycin-like cryptic lipopeptide encoded by *S. viridis*. Daptomycin has the simplest code: 4–33–33 333–33–333–33–3 333. In contrast, the code for the taromycin ACP differs from that of daptomycin at 15 positions. This may be due to the relatively short, di-unsaturated lipid starter processed by the taromycin FAAL:ACP (C8Δ2,4) versus the branched C12–13 lipids preferred by the daptomycin FAAL:ACP. Both couple fatty acids to l-Trp_1_ of these highly related tridecapeptides (Fig. 2b). The ACP code from the taromycin-like cryptic BGC from *S. viridis* differs from that of authentic taromycin at 12 positions, but only differs from the daptomycin code in 7 positions. This divergence pattern suggests that the cryptic BGC from *S. viridis* may encode initiation with a longer chain length, di-unsaturated fatty acid (see below). This could be tested by expressing the cryptic BGC in a *Streptomyces* expression host (Baltz, 2010a; Baltz, 2016; Xu & Wright, 2019).

**Table 4:** DptF (ACP) Multiprobe Codes

Microorganism	Lipopeptide (predicted)	DptF ACP homolog	ACP code
**Multiprobe sources**			
*S. roseosporus* NRRL 11379	Daptomycin	AAX31556	**4**-33-33333-33-333-33-3333
*S.* sp. CNQ490	Taromycin	WP_024877508	2-**44**-11122-32-322-33-1111
*S. viridis* DMS 43017	(desCl-Taromycin)	WP_082002416	2-**44**-23333-33-333-33-2232
*S. fradiae* A54145	A54145	AAZ23074	3-22-**44444**-33-332-32-1111
*S. exfoliates* SM41693	(A54145)	WP_037635382	3-23-**44444**-33-332-33-2223
*S. griseoluteus* ISP-5360	(A54145)	WP_051751218	3-22-**44444**-33-332-33-2223
*S. pini* PL19	(A54145)	WP_093851742	3-33-**44444**-33-332-33-1122
*S. barkulensis* RC 1830	(A54145)	WP_101254957	3-33-**44444**-33-332-33-2222
*A. friuliensis* DSM7358	Friulimicin	WP_023362358	3-33-33333-**44**-**444**-**44**-2223
*UncBac* GQ475284	Friulimicin	ADK54908	3-33-33333-**44**-**444**-**44**-3323
*S. viridochromogenes* ATCC29814	Laspartomycin^a^	AEF16024	3-33-33333-**44**-**444**-33-3333
*S. malaysiensis* DSM 4137	(Glycinocin)^a^	WP_099016109	3-33-33333-**44**-**444**-3**4**-3333
*S.* sp. 1331.2	(Glycinocin)	WP_097235610	2-33-22222-**44**-**444**-**4**3-3333
*UncBac* KY654519	Malacidin	ARU08072	3-33-33333-**44**-**4**33-**44**-3333
*UncBac* KF264539	(Malacidin)	AGS49326	3-33-33333-**44**-3**4**3-**44**-3333
*S. canus* ATCC 12646	Telomycin	AKQ13294	3-12-12111-22-333-33-**4444**
*S. canus* ATCC 12647	Telomycin	WP_059298593	3-12-12111-22-333-33-**4444**
*S. qaidamensis* S10	(Telomycin)	WP_062930001	3-12-12111-22-333-33-**4444**
*S. formicae* KY5	(Telomycin?)	WP_098240746	3-12-23322-33-333-33-**4444**
**Others**			
*S. fungicidicus* ATCC 21031	Enduracidin	ABD65955	3-33-23333-33-333-33-3333
*S.* sp. M56	Glycinocin	AUA09014	3-33-33333-**44**-**444**-3**4**-3333
*S. canus* ATCC 12237	Amphomycin	KUN68828	3-33-33333-**44**-**444**-33-3323
*S. parvulus* 2297	Parvuline?	RDD86143	3-33-33333-**44**-**444**-**44**-3333
*S. ambofaciens* ATCC 23877	(Lipotridecapeptide)	WP_053138555	3-33-23333-3**4**-**4**33-33-3333
*S. zhaozhouensis* CGMCC 4.4095	(Unknown)	SOD64425	3-22-12222-33-333-33-3333
*S. sedi* JMC 16909	(Unknown)	WP_139649588.1	3-**44**-33333-33-333-**44**-2223

^a^Laspartomycin is a member of the glycinocin family.

The ACP multiprobe codes for the A54145 BGC from *S. fradiae* and cryptic A54145 BGCs from *S. exfoliatis, S. griseoluteus, S. pini*, and *S. barkulensis* are closely related, but show some variation at 5 positions (Table 4). All of the variation resides in positions 2–3 (taromycins) and 16–19 (telomycins). The two friulimicin ACP codes differ from each other in positions 16 and 17 (telomycin). Other code differences within otherwise highly related BGCs may reflect differences in fatty acid chain length specificities (e.g., *S.* sp. 1331.2 and *S. formicae* KY5). These ACP codes are useful in identifying known, related, and novel lipopeptide BGCs (see below).

#### Fatty acid dehydrogenations

The cyclic lipopeptides have fatty acids ranging from C8 to C15 chain lengths. Some are unsaturated (e.g., daptomycin and A54145), and others have one or two double bonds. Laspartomycin has C15:Δ2; friulimicin C13–15:Δcis3; taromycin C8:Δ2,4; and malacidin C10–11:Δ2,4. The fatty acid chain length and degree of unsaturation can influence the biological activities of cyclic lipopeptide antibiotics, and are thus important targets for combinatorial biosynthesis as well as chemical semi-synthesis (Baltz et al., 2005; Baltz, 2014b, 2014d). FAAL and ACP genes are contiguous and just upstream of the first NRPS genes in the daptomycin, A54145, telomycin BGCs (Fig. 2). They are displaced by one or two genes (depicted as a space between FAAL and ACP genes in Fig. 2) in BGCs encoding lipopeptides with lipid side chains containing one or two double bonds. These genes encode enzymes that are annotated as acyl-CoA dehydrogenase family (ACAD). They encode enzymes that insert double bonds into the lipid starter units (Heinzelmann et al., 2005). Since there are no acyl-CoA intermediates involved in the FAAL:ACP:C^III^ lipopeptide initiation mechanism (Wittmann et al., 2008), it seems likely that these enzymes act on fatty acids bound to FAALs or to holo-ACPs, but no mechanistic studies have been reported for these enzymes.

Table 5 shows BLASTp analyses of the enzymes responsible for catalyzing the fatty acid dehydrogenations. LipB was demonstrated to carry out the Δcis3 dehydrogenation in friulimicin biosynthesis by gene disruption analysis (Heinzelmann et al., 2005). LipB has 60% sequence identity to the laspartomycin homolog Orf22 (Wang et al., 2011) that inserts the Δ2 double bonds. Both LipB and Orf22 share higher sequence identities with Tar5 and MlcH enzymes from the taromycin and malacidin pathways than to Tar6 and MlcI, the second fatty acid dehydrogenases encoded in the taromycin and malacidin BGCs (Yamanaka et al., 2014; Hover et al., 2018; Reynolds et al., 2018). Therefore, Tar5 and MlcH likely insert the Δ2 double bonds, and Tar6 and MlcI likely insert the Δ4 double bonds. Tar5 and Tar6 homologs are also encoded by the cryptic taromycin-like BGC in *S. viridis* (Table 5) (Baltz, 2010b).

**Table 5:** Acyl CoA Dehydrogenase Superfamily (ACAD) BLASTp Scores for Actinomycetes and Uncultured Bacteria

		Query protein^a^					
Actinomycete or uncultured bacterium	Fatty acid dehydrogenase	Tar5	MlcH	Fri (LipB)	Las (Orf22)	Tar6	MlcI
*Sa.* sp. CNQ490	Tar5	**100**	48	45	46	32	30
*Sa. viridis* DSM 43017	(Tar5)	**78**	50	46	48	31	30
*UncBac*^b^ KY654519	MlcH	48	**100**	53	50	34	30
*UncBac* KF264539	MlcH	50	**87**	53	52	33	30
*A. friuliensis* DSM 7358	LipA-Fri	46	53	**100**	60	31	29
*UncBac* GQ475284	LipA-Fri	45	54	**93**	60	32	28
*S. viridochromogenes* ATCC 29814	LipA-Las	46	51	60	**100**	32	27
*S. malaysiensis* DSM 4137	(LipA-Las/Gly)	46	51	59	**70**	30	29
*S.* sp. M56	(LipA-Las/Gly)	46	51	59	**70**	30	29
*S.* sp. 1331.2	(LipA-Las/Gly)	44	49	57	65	29	28
*S. canus* ATCC 12237	WP_059206888^b^	49	51	65	68	35	31
*S. parvulus* 2297	WP_114531150^b^	48	53	64	67	35	31
*S. ambofaciens* ATCC 23877	AKZ58687	51	54	50	51	35	29
*S. sedi* JMC 16909	WP_139649592	54	50	48	46	35	-
*Sa.* sp. CNQ490	Tar6	32	34	31	30	**100**	48
*Sa. viridis* DSM 43017	(Tar6)	33	33	34	31	**78**	48
*UncBac* KY654519	MlcI	30	30	29	27	48	**100**
*UncBac* KF264539	MlcI	29	29	28	27	46	**90**
*S. ambofaciens* ATCC 29814	AKZ58688	31	35	31	31	50	56
*S. sedi* JMC 16909	WP_139649590	33	36	29	34	52	48

^a^Possible orthologs are shown in bold.

^b^These proteins share 78% sequence identities.

Tar5 and Tar6 paralogs share 32% sequence identities, and MlcH and MlcI share 30%. Even though Tar5 and MlcH, and Tar6 and MlcI appear to have similar functions, they have diverged substantially, presumably to accommodate different fatty acid chain length preferences, and the associated divergences in FAAL and ACP amino acid sequences (Tables 2–4). These proteins can be used in conjunction with other molecular beacons to analyse lipopeptide BGCs for similarities and novelty (see below).

### Cyclization and Release (Termination)

#### Dimodular termination devices

A second important biosynthetic device for lipopeptide assembly is the dimodular NRPS with a CAT-CATTe organization for termination and release of completed lipopeptides (Fig. 2a). When combinatorial biosynthetic studies were initiated at Cubist Pharmaceuticals in the early 2000s, only three cyclic lipopeptide BGC sequences were available, those for daptomycin (Miao et al., 2005), A54145 (Miao, Brost, et al., 2006), and CDA (Hojati et al., 2002). These BGCs were chosen because they appeared to by evolutionarily related, as witnessed by conserved amino acid chirality in the ten-membered rings and in the conservation of dimodular NRPS genes that inserted 3mGlu-Kyn, 3mGlu-Ile, and 3mGlu-Trp, respectively (Miao, Coëffet-Le Gal, et al., 2006). All three NRPS genes also encoded terminal Te domains. This type of NRPS dimodule is also used for biosynthesis of friulimicin/laspartomycin type lipopeptides (Fig. 2) (Müller et al., 2007; Wang et al., 2011). With recent publications of telomycin (Fu et al., 2015; Johnston et al., 2016; Liu et al., 2016) and malacidin BGC sequences (Owen et al., 2013; Hover et al., 2018), it is now apparent that the dimodular CAT-CATTe mechanism for termination of lipopeptide assembly is used among these structurally diverse lipopeptide BGCs, and likely derives from very ancient origins (Baltz, 2010b). Table 6 shows the sequence identities among the eight DptD homolog types. The taromycin, A54145, CDA, friulimicin, telomycin, and malacidin clades show at least 80% sequence identities to likely orthologs within the clades, and less than 60% identities to paralogs in other clades. The apparent laspartomycin/glycinocin clade shows over 70% sequence identities within the clade. Interestingly, DptD shows less than 60% sequence identity with the two members of the taromycin clade, in spite of the fact that they all have CAT-CATTe dimodules that insert 3mGlu_12_-Kyn_13_ or 3mGlu_12_-4clKyn_13_ into nearly identical lipopeptide tridecapeptides (Fig. 2b). This divergence may be due to the substantial differences in fatty acid structures that need to be accommodated during thioesterase cyclization and release (termination).

**Table 6:** DptD NRPS (CAT-CATTe) BLASTp Scores in Select Actinomycetes and Uncultured Bacteria

		Query protein^a^							
Actinomycete or uncultured bacterium	DptD homolog (predicted)	DptD	Tar10	LptD	PSIII	PstD	LpmD	Tem22	MlcM
*S. roseosporus* NRRL 11379	DptD	**100**	55	53	52	49	48	51	46
*Sa.* sp. CNQ490	Tar10	56	**100**	50	50	47	47	49	45
*Sa. viridis* DSM 43017	(DptD)	56	**82**	50	51	48	48	47	46
*S. fradiae* A54145	LptD	53	50	**100**	50	49	49	46	46
*S. exfoliates* SM41693	(LptD)	53	50	**93**	50	49	49	46	46
*S. griseoluteus* ISP-5360	(LptD)	53	50	**93**	50	49	49	46	46
*S. pini* PL19	(LptD)	52	50	**81**	50	48	48	47	46
*S. barkulensis* RC 1830	(LptD)	52	50	**81**	49	48	49	46	46
*S. coelicolor* A3(2)	PSIII	52	50	50	**100**	49	50	49	48
*S. lividans* TK24	PSIII	52	50	50	**99**	49	50	48	48
*S.* sp. MBT28	(PSIII)	52	50	50	**90**	49	50	48	48
*S.* sp. NRRL WC-3795	(PSIII)	49	46	48	**90**	49	50	49	46
*A. friuliensis* DSM 7358	PstD	49	47	49	49	**100**	64	54	51
*UncBac* GQ475284.1	PstD	48	47	49	49	**93**	64	53	51
*S. viridochromogenes* ATCC 29814	LpmD	49	47	49	50	64	**100**	54	51
*S. malaysiensis* DSM 4137	(LpmD}	50	49	49	51	65	**77**	55	51
*S.* sp. M56	(LpmD)	50	49	49	51	65	**77**	55	51
*S.* sp. SPMA113	(LpmD)	50	49	49	51	65	**77**	55	51
*S.* sp. 1331.2	(LpmD)	49	47	49	51	65	**73**	54	52
*S. canus* ATCC 12646	Tem22	48	49	48	48	54	54	**100**	48
*S. canus* ATCC 12647	Tlo22 = Tem22	48	48	48	48	54	54	**98**	48
*S. qaidamensis* S10	(Tem22}	48	47	46	48	53	53	**95**	49
*S. formicae* KY5	(Tem22)	48	46	45	48	52	52	**80**	48
*UncBac* KY654519.1	MlcM	47	45	46	48	51	51	48	**100**
*UncBac* KF264538.1	(MlcM)	47	45	46	47	51	51	49	**86**
*S. canus* ATTC 12237	WP_059209689^b^	51	48	50	52	67	74	54	53
*S. parvulus* 2297	WP_114529432^b^	49	49	50	52	67	72	55	53
*S. ambofaciens* ATCC 23877	AKZ258685	47	47	46	48	47	47	47	45
*S. zhaozhouensis* CGMCC 4.7095	SOD64429	53	51	48	51	50	50	48	46
*S. sedi* JCM 16909	WP_139647437	65	57	54	53	49	49	48	47

^a^Possible orthologs are shown in bold.

^b^These proteins share 86% sequence identities.

#### Amino acid binding pocket analysis of dimodular termination devices

The amino acid binding pockets in A domains determine which amino acids are bound, activated, and incorporated during peptide assembly (Challis et al., 2000; Stachelhaus et al., 1999). Table 7 shows that phylogenetic relationships between amino acid binding pockets in CAT-CATTe didomains can be used to help distinguish between known, related, and novel lipopeptides (see below). Daptomycin, A54145, and CDA have 3mGlu incorporated at position one of NRPS termination didomains, and Kyn, Ile/Val, and Trp at position two. There are three related binding codes for 3mGlu: DLGKTGVINK for daptomycin; DLGKTGVVNK for two taromycins and five A54145s; and DQGGKTGVGHK for four CDAs. The daptomycin DptD module-1 differs from those of two taromycins and five A54145s by single conserved change at position 8 of the pocket (I for V). The CDA 3mGlu pocket differs from those of daptomycin, taromycin, and A54145 at positions 2, 8, and 9. For module-2, Kyn has two pocket codes: DAWTTTGVGK for daptomycin and cryptic taromycin from *S. viridis*; and DAWTTTGVAK for taromycin from S*accharomonospora* sp. CNQ490. These differ by a conserved substitution at position 9 (G or A). All A54145 module-2 pockets for Ile/Val are identical (DGLFVGIAVK), as are all CDA module-2 pockets for Trp (DGWAVASVCK). The friulimicin, laspartomycin/glycinocin, and malacidin lipopeptide families have termination dimodules that insert Val-Pro (Fig. 2). Among them, they use four different, but somewhat related amino acid binding codes for insertion of Val, and four different, but related codes for Pro. The telomycin termination dimodule inserts Ile-Pro. The Ile binding code is identical for four Tem22 orthologs, but is substantially different from the Val modules of friulimicins, laspartomycin/glycinocins, and malacidin, and the Ile/Val modules of A54145s. The amino acid binding codes for these lipopeptide dimodules establishes a baseline to help triage and characterize other known, related, and novel lipopeptide BGCs from finished and draft genome sequences (see below).

**Table 7:** DptD Homolog Binding Pockets in Select Actinomycetes and Uncultured Bacteria

Actinomycete or uncultured bacterium	DptD homolog (predicted)	Binding pocket amino acid 1	Specificity amino acid 1	Binding pocket amino acid 2	Specificity amino acid 2
*S. roseosporus* NRRL 11379	DptD	DLGKTGVINK	3mGlu	DAWTTTGVGK	Kyn
*Sa.* sp. CNQ490	Tar10	DLGKTGVVNK	3mGlu	DAWTTTGVAK	Kyn
*Sa. viridis* DSM 43017	(Tar10)	DLGKTGVVNK	3mGlu	DAWTTTGVGK	Kyn
*S. fradiae* A54145	LptD	DLGKTGVVNK	3mGlu	DGLFVGIAVK	Ile/Val
*S. exfoliates* SM41693	(LptD)	DLGKTGVVNK	3mGlu	DGLFVGIAVK	Ile/Val
*S. griseoluteus* ISP-5360	(LptD)	DLGKTGVVNK	3mGlu	DGLFVGIAVK	Ile/Val
*S. pini* PL19	(LptD)	DLGKTGVVNK	3mGlu	DGLFVGIAVK	Ile/Val
*S. barkulensis* RC 1830	(LptD)	DLGKTGVVNK	3mGlu	DGLFVGIAVK	Ile/Val
*S. coelicolor* A3(2)	PSIII	DQGKTGVGHK	3mGlu	DGWAVASVCK	Trp
*S. lividans* TK24	PSIII	DQGKTGVGHK	3mGlu	DGWAVASVCK	Trp
*S.* sp. MBT28	(PSIII)	DQGKTGVGHK	3mGlu	DGWAVASVCK	Trp
*S.* sp. NRRL WC-3795	(PSIII)	DQGKTGVGHK	3mGlu	DGWAVASVCK	Trp
*A. friuliensis* DSM 7358	PstD	DAYWWGGTFK	Val	DVQYVAHVVK	Pro
*UncBac* GQ475284.1	PstD	DAYWWGGTFK	Val	DVQYVAHVVK	Pro
*S. viridochromogenes* ATCC 29814	LpmD	DAYFWGVCFK	Val	DVQYIAHVVK	Pro
*S. malaysiensis* DSM 4137	(LpmD}	DAYFWGVTFK	Val	DVQYVAHVVK	Pro
*S.* sp. M56	(LpmD)	DAYFWGVTFK	Val	DVQYVAHVVK	Pro
*S.* sp. SPMA113	(LpmD)	DAYFWGVTFK	Val	DVQYVAHVVK	Pro
*S.* sp. 1331.2	(LpmD)	DAYWWGGTFK	Val	DVQYVAHVVK	Pro
*S. canus* ATCC 12646	Tem22	DAYLTGLMNK	Ile	DVQFVSQVMK	Pro
*S. canus* ATCC 12647	Tlo22 = Tem22	DAYLTGLMNK	Ile	DVQFVSQVMK	Pro
*S. qaidamensis* S10	(Tem22}	DAYLTGLMNK	Ile	DVQFVSQVMK	Pro
*S. formicae* KY5	(Tem22)	DAYLTGLMNK	Ile	DVQFVAQVVK	Pro
*UncBac* KY654519.1	MlcM	DAFWLGGTFK	Val	DVQYVGHAIK	Pro
*UncBac* KF264538.1	(MlcM)	DAFWLGGTFK	Val	DVQYVGHAIK	Pro
*S. canus* ATTC 12237	WP_059209689	DAYWWGGTKF	Val	DVQYVAHVVK	Pro
*S. parvulus* 2297	WP_114529432	DAYWWGGTFK	Val	DVQYVAHVVK	Pro
*S. ambofaciens* ATCC 23877	AKZ258685	DFWNVGMVHK	Thr	DIYHLGLLCK	Hpg
*S. zhaozhouensis* CGMCC 4.7095	SOD64429	DHTKIGAITKINK	Asp	DAWTTTGVAK	Kyn
*S. sedi* JCM 16909	WP_139647437	DLGKTGVINK	3mGlu	DAWTTTGVGK	Kyn

### Activation and Sequential Coupling of Amino Acids to Amino Acids (Elongation)

Sandwiched between the initiation and termination devices for lipopeptide assembly are the elongation devices of variable composition. These are NRPS proteins that generally contain multiple modules to catalyze sequential amino acid couplings. The elongation process provides a fertile evolutionary “workshop” to test different combinations of amino acids and peptide lengths with varying chirality for activities that impart survival advantages for the producing microorganisms. The evolutionary changes in primary amino acid sequence can be coupled with modifications of fatty acid chain length and degree of unsaturation, and amino acid modifications as discussed below. Some of these NRPS multimodular proteins can be used to confirm known BGC types, and to identify new and novel BGCs in finished BGCs (see below). Because of their generally large sizes with repetitious functional domains, they are often misassembled in draft genomes (Baltz, 2017b; Baltz, 2019; Goldstein et al., 2019; Klassen & Currie, 2012), and generally not suitable to use as primary molecular beacons.

### Amino Acid Modifications

During the course of lipopeptide pathway evolution, changes in amino acid composition have occurred. In some cases, these include amino acid modifications. Aside from the many examples of the use of d-amino acids, additional examples are the inclusion of 3mGlu in daptomycin, taromycin, A54145, and CDA; hAsn in A54145 and CDA; moAsp in A54145; hAsp in malacidin; mGly (Sar) in A54145; mTrp in telomycin; and mAsp in malacidin, friulimicin, amphomycin, and parvuline (Baltz et al., 2005; Fu et al., 2015; Johnston et al., 2016; Hover et al., 2018) (Fig. 2). These are important for subtle alterations in biological activity and can be manipulated for combinatorial biosynthesis by simple gene deletions, as demonstrated in combinatorial manipulations of the A54145 pathway to generate highly active antibiotics with improved properties (Nguyen et al., 2010; Alexander et al., 2011; Baltz, 2014b). Several of these amino acid modifying enzymes have been used as molecular beacons for use in genome mining (Supplementary Table S1), and can be used to help triage known and novel BGCs (Baltz, 2010b, 2018) (see below).

### MbtH Chaperones

Many NRPS-based BGCs include *mbtH* homologs that encode small nonenzymatic chaperones that enhance certain adenylation reactions (Baltz, 2011; Baltz, 2014a). MbtH homologs have diverged substantially in different NRPS BGCs, and can be considered as orthologs, paralogs, or “ortho-paralogs,” proteins with similar functions but different protein-protein interactions (Baltz, 2018). Among the MbtH homologs, a high degree of sequence similarity is observed within BGCs encoding similar products, but much higher sequence divergence is observed between MbtH homologs from unrelated BGCs (Baltz, 2014a) Supplementary Table S3 shows the BLASTp scores between different MbtH homologs from lipopeptide BGCs. MbtH apparent orthologs show >80% sequence identities within BGC clades, and relatively high sequence identities between lipopeptide clades.

The degree of MbtH divergence can also be assessed by BLASTp analysis with a 24-mer multiprobe consisting of the most conserved 60 amino acid segments from 24 diverse MbtH homologs (Baltz, 2014a). Supplementary Table S4 shows the MbtH multiprobe codes for lipopeptide BGCs. The 24 digit numerical codes are highly related, with a consensus of 332–333–322–333–322–222–223–312. Of the 16 MbtH homologs analysed, five have consensus sequences (one taromycin, two CDAs, one laspartomycin, and one telomycin), 7 deviate at 1 position, 3 at 2 positions, and 1 at 4 positions, giving an average deviation from consensus of 4.4%. Supplementary Table S5 shows the MbtH consensus codes for six diverse NRPS BGC families. The bleomycin family includes tallysomycin and zorbamycin BGCs, and the five producers span four actinomycete genera; the five griseobactin MbtH homologs are encoded by *Streptomyces* sp.; the two nikkomycin BGCs are encoded by *Streptomyces* sp.; the two nocardicin A BGCs are encoded by *Nocardia uniformis* and *Actinosynnema mirum*; and the pacidamycin family includes napsamycin and sansanmycin BGCs from *Streptomyces* sp. (Baltz, 2014a). The deviations from consensus within families range from 1.7% for griseobactin to 6.9% for the bleomycin family (Supplementary Table S5). The consensus MbtH codes for the five diverse BBG families deviate from that of the lipopeptide family by ∼70–95%. The exception is the glycopeptide consensus codes that differ by only 8%. These data suggest that the MbtH-like chaperone function has been conserved in the lipopeptide family of BGCs, and indicate that BLASTp analysis with individual MbtH homologs and the MbtH multiprobe can be used in conjunction with other molecular beacons to identify genomes encoding known, related, and novel lipopeptides. The MbtH multiprobe can also triage other more distantly related NRPS BGCs, and help identify novel BGCs encoded in finished or draft actinomycete genomes (Baltz, 2014a).

## Resistance and Transport

Incremental resistance to daptomycin in low G + C pathogenic Gram^+^ bacteria is mediated by mutations in a number of genes that result in alterations in cell membrane charge or cell wall thickness (Baltz, 2009, 2014d). None of the genes involved in these mechanisms are observed in lipopeptide BGCs. The high G + C Gram^+^ actinomycetes tend to be intrinsically resistant to daptomycin by expressing hydrolases that cleave the fatty acid tail, or the depsipeptide bond, and many show secondary peptide bond cleavages (D'Costa et al., 2006, 2012; Baltz, 2014d). These mechanisms often result in MICs > 256 μg/ml. Genes encoding these hydrolase mechanisms are not observed in lipopeptide BGCs. Instead, it is likely that resistance to lipopeptide antibiotics in the producing microorganisms is mediated by transport mechanisms, as accumulation of high intracellular lipopeptide concentrations could be highly toxic.

The daptomycin BGC in *S. roseosporus* encodes an ABC transporter, including an ATP-binding cassette protein (DptM) and transmembrane permease (DptN). Another gene (*dptP*) is clustered with *dptM* and *dptN* (Miao et al., 2005). These three genes are located just upstream of the FAAL, ACP, NRPS cluster, *dptEFABCD. dptMNP* homologs are also located upstream from *dptEFABCD* homologs in *Saccharomonospora* strains that encode taromycin (Yamanaka et al., 2014; Reynolds et al., 2018) and cryptic des-chloro-taromycin (Baltz, 2010b, 2018), and downstream of the A54145 BGC genes *lptEFABCDGHJKL* in *S. fradiae* (Miao, Brost, et al., 2006). All of the other lipopeptide BGCs contain two genes encoding ABC transporters, but lack *dptP* homologs. Supplementary Tables S6, S7, and S8 show the phylogenetic relationships between DptM, DptN, and DptP homologs.

The DptM, DptN, and DptP proteins showed >70% sequence identities within individual clades. DptM and DptN homologs from the taromycin, A54145, friulimicin, laspartomycin and malacidin pathways showed >50% sequence identities in pairwise BLASTp analyses, but DptMN homologs showed only 29–34% sequence identities (DptM) or no sequence identities (DptN) with those of CDA and telomycin pathways. The DptM and DptN ABC transporter counterparts from the CDA and telomycin pathways showed 65–66% (DptM-like) and 41–42% (DptN-like) identities with each other. This suggests that two distinct lines of evolution have contributed to the ABC transporters for lipopeptide antibiotics. DptP is interesting in that it is found only in daptomycin/taromycin and A54145 BGCs, and that DptP from the daptomycin BGC shows 91% sequence identity with LptP from the A54145 pathway, suggesting possible horizontal gene transfer. When DptP was integrated into the chromosome of *S. ambofaciens*, an unusual streptomyces susceptible to daptomycin, the recombinant strain became resistant to daptomycin (Baltz, 2008b), suggesting that DptP may normally interact with DptMN to export daptomycin, and may interact with a close homologs of DptMN in *S. ambofaciens* to express the DapR phenotype (see below).

From an evolutionary perspective, the general use of a molecular target agnostic ABC transporter mechanism for resistance and export of lipopeptide antibiotics facilitates natural combinatorial biosynthesis of molecules with different target specificities and mechanisms of action (MOA), as is the case for cyclic lipopeptide antibiotics (Baltz, 2009; Johnston et al., 2016; Hover et al., 2018). This aspect of lipopeptide BGCs should facilitate successful combinatorial biosynthesis of compounds with improved antibacterial properties and toxicity profiles, and possible beneficial changes in MOA, without jeopardizing the viability of the recombinants producing the new molecules. This may help explain the high success rates of producing novel lipopeptides by combinatorial biosynthesis at Cubist Pharmaceuticals (Baltz, 2014b, 2014c, 2014d). So far, little is known about the substrate specificities and possible cross-resistance patterns expressed by lipopeptide ABC transporters.

## Use of Molecular Beacons for Genome Mining

Over 2 300 genome sequences from filamentous actinomycetes were publically available on the NCBI website (https://www.ncbi.nlm.nih.gov/genome ) in July of 2020. A large majority (∼90%) are in draft form, which is problematic for the identification of complete lipopeptide BGCs because of frequent misassembly of multimodular NRPS genes (Klassen & Currie, 2012; Baltz, 2017b, 2019; Goldstein et al., 2019). To accommodate productive genome mining of both finished and draft genomes, two strategies can be implemented. Gifted microorganisms (Baltz, 2014a, 2017a, 2017b) can be surveyed for the presence of genes (molecular beacons) directed at conserved functions by BLASTp to identify strains encoding targetted SM-BGC classes. Supplementary Table S1 summarizes examples of molecular beacons for lipopeptide antibiotics, some of which have been used previously, and others exemplified here. For finished genomes, molecular beacon analysis can be followed directly by antiSMASH (5.0 at the time of this writing) to identify draft lipopeptide BGCs, and to predict the NRPS subunits, amino acid binding specificities, gene composition and BGC organization. For unfinished genomes, the most promising microorganisms can be identified by molecular beacon analysis, sequenced to completion, the analysed by antiSMASH analysis of the targeted BGCs.

### Identification of common lipopeptide BGCs

Many cyclic acidic lipopeptide antibiotics were reported in the 1950s and 1960s, so it is likely that their BGCs will dominate the molecular beacon analyses because of their relatively high natural abundance. Examples include amphomycin, aspartamycin, laspartamycin/glycinocin, friulimicin, parvuline, tsushimycin, zaomycin, glutamycin, and related lipopeptides (Baltz et al., 2005). Of these, the BGCs of only two (friulimicin and laspartomycin) have been reported (Müller et al., 2007; Wang et al., 2011). Friulimicin and laspartomycin have two fundamental biosynthetic features in common with daptomycin, taromycin, A54145, telomycin, and malacidin: FAAL:ACP for initiation; and CAT-CATTe di-modules for termination (Fig. 2). They also have unique features that distinguish them from other lipopeptide BGCs: trans NRPS tri-domain (ATTe) proteins, PstA and LpmA, that interact with the CTs imbedded in tri-modular NRPS (CAT-CT-CATE) proteins, PstB and LptB. This mechanism is also used by the more recently discovered malacidin (Hover et al., 2018) (Fig. 2). The PstA and LpmA proteins are small and lack repetitive domains, so it is likely that *pstA* and *lpmA* homologs will be assembled correctly in draft genomes. Friulimicin, and closely related molecules, amphomycin, parvuline, and tsushimycin, insert 3mAsp just upstream of the Asp-Gly-Asp-Gly Ca^2+^-binding sequence (Baltz et al., 2005) (Fig. 2). In the friulimycin producer, *A. friuliensis*, 3mAsp is biosynthesized by the two subunit glutamate mutase, GlmA/GlmB (Heinzelmann et al., 2003). Thus PstA/LpmA and GlmA/GlmB are useful as molecular beacons to triage known lipopeptide BGCs. One example is *S. canus* ATCC 12237, a known producer of amphomycin (Baltz et al., 2005). The amphomycin peptide differs from friulimicin at position 1 (l-Asp vs. l-Asn in friulimicin) (Baltz et al., 2005). BLASTp analysis of the draft genome of *S. canus* ATCC 12237 indicated that it encodes FAAL, ACP, and CAT-CATTe NRPS enzymes (Tables 2, 3, and 6), and has an ACP multiprobe code similar to those of friulimicin/laspartomycin/glycinocin (Table 4). It encodes GlmA and GlmB homologs needed for biosynthesis of 3mAsp (not shown), and a LipB homolog needed to insert the Δ3 double bond in the fatty acid (Table 5). It has a typical lipopeptide MbtH homolog with high sequence similarity to those of the friulimicin and laspartomycin/glycinocin BGCs (Supplementary Tables S3 and S4), and an ABC transporter most closely related to that of laspartomycin (Supplementary Tables S6 and S7). It has a PstA (ATTe) homolog (Supplementary Table S9), but PstB is missing, likely due to inadequate assembly of this larger NRPS in the draft genome. Importantly, its DptD homolog termination dimodule has amino acid specificity for Val-Pro (Table 7). The Val binding code is identical those of friulimicins, and the Pro binding code is identical to those of several in the laspartomycin/glycinocin group. This strain of *S. canus* is a good candidate for finished genome sequencing to provide a complete amphomycin BGC for the MiBIG database (Kautsar et al., 2020).

*Streptomyces parvulus* NRRL 5740 was reported to produce parvuline, a member of the amphomycin family (Baltz et al., 2005). Its peptide is identical to that of amphomycin, and it has a Δ3-iso-deceneoyl fatty acid side chain rather than the Δ3-anteiso-decenoyl side chain observed in amphomycin (Baltz et al., 2005). Thus the BGCs of parvuline and amphomycin should be highly homologous. The draft genome of the recently isolated *S. parvulus* 2297 (Hu et al., 2018) contains many genes required for parvuline biosynthesis (Tables 2, 3, 5, 6, Supplementary Tables S3, S4, and S6–S9). Its ACP multiprobe code places its BGC within the friulimicin/laspartomycin/glycinocin group of related lipopeptides (Table 4). As anticipated, the apparent parvuline biosynthetic proteins show the highest sequence similarities (78–90%, average 85%) to the apparent amphomycin biosynthetic proteins encoded in *S. canus* ATCC 12237 (Tables 2, 3, 5, 6, Supplementary Tables S3, S6, S7, and S9). Notably, the DptD homolog termination dimodule inserts Val-Pro, and the binding codes are identical to those of the predicted amphomycin BGC from *S. canus* ATCC 12237. The large NRPS genes involved in lipopeptide elongation are not assembled correctly, so it would be useful to obtain a finished genome to add a parvuline BGC to the MiBIG database (Kautsar et al., 2020). The molecular beacons used in these two examples can be used to triage common lipopeptide BGCs that encode molecules of little current interest for drug development, and to help focus on those with higher potential for clinical development.

### Identification of Lipopeptide BGCs Related to Important Clinical Antibiotics

Daptomycin is an important antibiotic approved to treat difficult to treat Gram-positive infections, including MRSA (Baltz, 2009; Eisenstein et al., 2010). Daptomycin has 3mGlu at position 12 in the peptide. 3mGlu is biosynthesized by a mechanism that employs an α-ketoglutarate methyltransferase (Milne et al., 2006) encoded by *dptI*, a gene that has distantly related homologs in the A54145 (*lptI*) and CDA (*glmT*) BGCs. DptI, LptI, and GlmT are useful molecular beacons to identify lipopeptide BGCs containing the rare 3mGlu, and for sorting them into daptomycin, A54145, and CDA related clades (Baltz, 2018). DptI was used in such a search over a decade ago, and it led to the discovery of a DptI homolog (DptI-sv) in *S. viridis* imbedded in a cryptic lipopeptide BGC closely related to that of daptomycin (Baltz, 2010b). More recently the taromycin BGC, closely related to the cryptic BGC in *S. viridis*, was cloned from the marine *Saccharomonospora* sp. CNQ490 and expressed in *S. coelicolor* (Yamanaka et al., 2014; Reynolds et al., 2018). The taromycin BGC encodes a DptI homolog, Tar13. DptI was used in a recent BLASTp search and identified another DptI homolog (DptI-ss), encoded by *Streptomyces sedi* JCM 16909 (Table 8). DptI-ss is more closely related to DptI, DptI-sv, and Tar13 than to any of the LptI or GlmT apparent orthologs, suggesting that it may be involved in the biosynthesis of a daptomycin-like lipopeptide. The draft genome of *S. sedi* (Li et al., 2009) also encodes a dimodular termination NRPS that shows 65% sequence identity to DptD, and has amino acid binding codes for 3mGlu-Kyn identical to those of DptD (Table 7). It has lipopeptide initiation FAAL and ACP homologs that are 56% and 50% identical to DptE and DptF (Tables 2 and 3), and an ACP multiprobe code similar to, but differing from that of daptomycin at five positions (Table 4). It encodes two ACAD-family fatty acid dehydrogenases distantly related to those of the taromycin BGC, suggesting that it initiates lipopeptide biosynthesis with a di-unsaturated fatty acid, perhaps of longer chain length than that of taromycin. *S. sedi* encodes a DptG homolog in the lipopeptide family, and the MbtH multiprobe analysis indicates that it differs from that of daptomycin at two positions, and taromycin at one position (Supplementary Table S3 and S4). *S. sedi* encodes an ABC transporter pair that shows 65 and 70% sequence identities to DptM and DptN, respectively, but no DptP homolog (Supplementary Tables S6–S8). The *S. sedi* genome sequence includes several NRPS fragments that show >60% sequence identities to portions of the two large NRPS proteins involved in daptomycin biosynthesis, DptA and DptBC (not shown). *S. sedi* is a prime candidate to obtain a finished genome sequence to determine if it encodes a new lipopeptide antibiotic related to daptomycin and taromycin.

**Table 8:** DptI Homolog BLASTp Scores in Actinomycetes

		Query protein^a^				
Actinomycete	DptI homolog	DptI	Tar13	(DptI-ss)	LptI	GlmT
*Streptomyces roseosporus*	DptI	**100**	**56**	**62**	38	37
*Saccharomonospora* sp. CNQ490	Tar13	**55**	**100**	**60**	38	38
*Sa. viridis* DSM 43017	(DptI-sv)^b^	**54**	**82**	**59**	39	40
*S. sedi*	(DptI-ss)	**62**	**60**	**100**	42	38
*S. fradiae* A54145	LptI	38	38	42	**100**	32
*S. exfoliates*	(LptI)	38	38	40	**87**	34
*S. griseoluteus*	(LptI)	37	40	40	**87**	35
*S. pini*	(LptI)	41	41	41	**75**	39
*S. barkulensis*	(LptI)	46	44	44	**75**	43
*S. coelicolor*	GlmT	37	39	38	32	**100**
*S. lividans*	GlmT	37	39	38	32	**100**
*S.* sp. MT28	(GlmT)	37	40	36	36	**89**
*S.* sp. NRRL WC-3795	(GlmT)	38	40	37	35	**90**

^a^Proteins most highly related to each other are shown in bold.

^b^( ), assigned bioinformatically. All others assigned functionally, and supported by chemical structures.

### Identification of Novel BGCs from Finished and Draft Genomes

#### Finished genomes

**Fig. 3:**
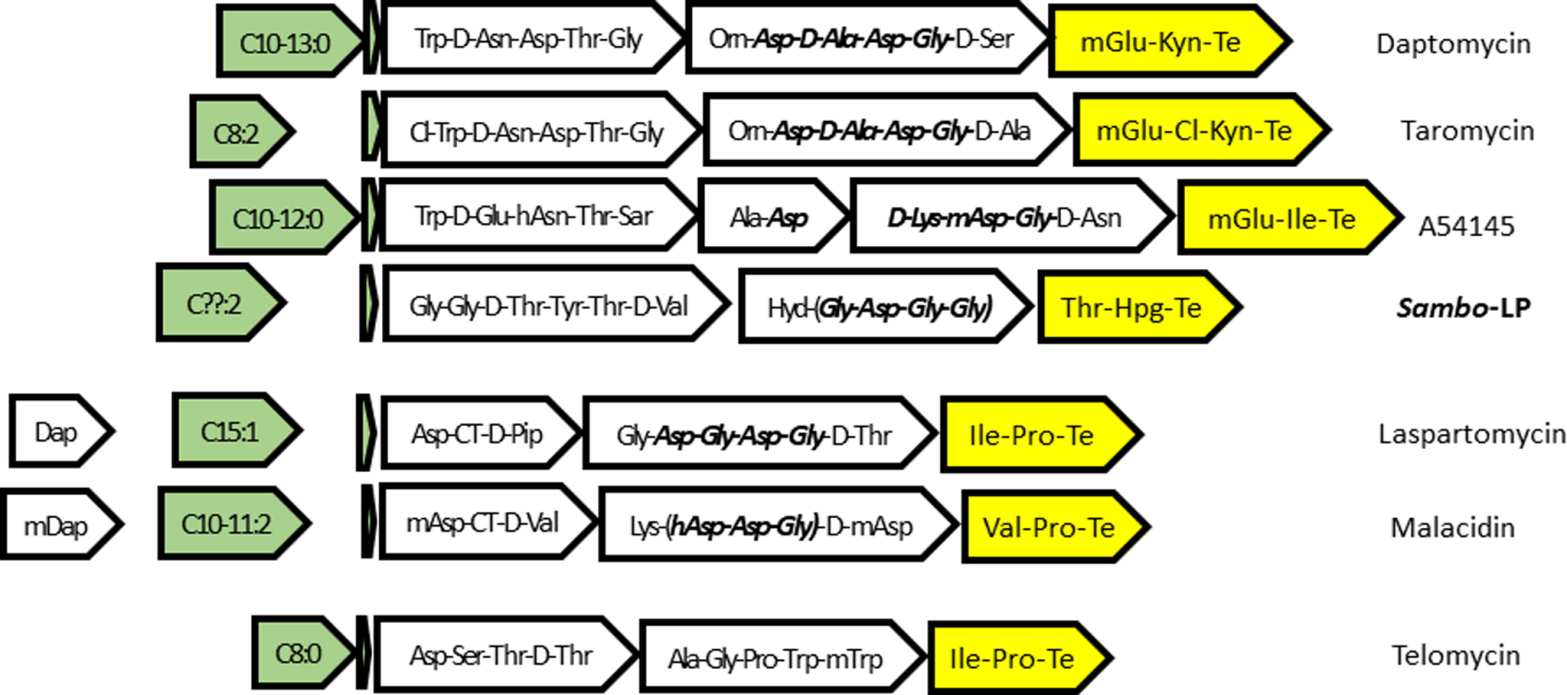
Organization of lipopeptide BGC genes involved in initiation, elongation, and termination of a cryptic lipopeptide encoded by *S. ambofaciens* (***Sambo-*LP**). The gene organization and NRPS subunit structures are distantly related to those of daptomycin, taromycin, and A54145, all of which encode tridecapeptides. The amino acid binding specificities differ substantially, however. The Ca^2+^-binding tetrapeptides of daptomycin, taromycin, A54145, laspartomycin are shown in bold italics. The amino acid regions that could be converted to canonical Ca^2+^-binding sites by insertion of a single Asp or Gly module for *Sambo*-LP or malacidin, respectively, are shown in bold italics in parentheses.

BLASTp analysis of the nonredundant bacterial sequences in NCBI indicated that the finished genome of *Streptomyces ambofaciens* (Aigle et al., 2014; Thibessard et al., 2015) encodes a DptD homolog with <50% sequence identity to any of the known lipopeptide dimodular termination NRPSs (Table 6). Its DptD homolog has amino acid binding specifities for Thr-Hpg (Table 7). BLASTp analysis indicated that it encodes FAAL and free-standing ACP enzymes for initiation of lipopeptide assembly not closely related to any of the known cyclic lipopeptides (Tables 2 and 3). The ACP multiprobe code is consistent with a lipopeptide assignment, but differs from those of known lipopeptide ACPs (Table 4). antiSMASH 5.0 analysis indicates that a lipopeptide BGC abuts the PKS-I BGC encoding the 16-membered macrolide antibiotic spiramycin. These two BGCs cluster with two other SM-BGCs, and are located in the core region containing mostly primary metabolic genes (Aigle et al., 2014). The lipopeptide BCG contains three NRPS genes composed of 6, 5, and 2 modules (Fig. 3), and is predicted to encode a tridecapeptide. The d-thr in position three could be involved in the formation of a depsipeptide bond to form a cyclic peptide. The novel lipopeptide BGC also encodes two ACAD-family fatty acid dehydrogenases, suggesting that lipopeptide assembly is initiated with a di-unsaturated fatty acid. It also encodes DptM and DptN ABC transporter homologs most closely related to those of daptomycin, taromycin, and A54145, and a DptG homolog in the lipopeptide MbtH family (Supplementary Tables S3–S5). It does not encode a DptP homolog. It is conceivable that the DptMN homolog ABC transporter interacts with heterologously expressed DptP in *S. ambofaciens* to express resistance to daptomycin (see above). This novel lipopeptide lacks a canonical Ca^2+^-binding tetrapeptide, but is one module insertion away in the Gly-Asp-Gly-Gly region of modules 8–11, as is malacidin between modules 5 and 6 (Fig 3). This cryptic lipopeptide BGC is a prime candidate for homologous or heterologous expression studies to assess biological activity, as it may provide a new scaffold for modification by combinatorial biosynthesis and medicinal chemistry.

#### Draft genomes

The draft genome sequence of *Streptomyces zhaozhouensis* (He et al., 2014) has a *dptD* homolog encoding a lipopeptide termination protein that shows 46–53 sequence identities to DptD homologs. Amino acid binding pocket analysis indicates that it incorporates Asp-Kyn. The Kyn binding pocket utilizes the same code as taromycin (Table 7). To my knowledge, this is the first instance of the use of Kyn in a termination dimodule differing from 3mGlu-Kyn of the daptomycin family of lipopeptides (daptomycin, taromycins, and the lipopeptide encoded by *S. sedi* reported here). *S. zhaozhouensis* also encodes DptE and DptF homologs not closely related to those of known lipopeptides (Tables 2 and 3), and the ACP multiprobe code is unique (Table 4). It does not encode an ACAD-family fatty acid dehydrogenase, suggesting that it initiates lipopeptide biosynthesis with an unsaturated fatty acid. It encodes no DptI homolog, consistent with its insertion of Asp-Kyn. It has an ABC transporter pair most closely related to those of CDA and telomycin (Supplementary Tables S6 and S7), giving additional credence to two lines of evolution of ABC transporters for lipopeptide BGCs. The predicted BGC also lacks a *dptP* gene. The combined information indicates that *S. zhaozhouensis* encodes a novel lipopeptide. As such, *S. zhaozhouensis* is a good candidate for finished genome sequencing and further analysis to assess biological activity and suitability for further SAR studies. Alternatively, the lipopeptide BGC could be cloned and sequenced by traditional methods.

## Discussion

Biosynthesis of lipopeptides related to daptomycin is carried out by a mechanism reminiscent of protein biosynthesis; it can be described in terms of initiation, elongation, and termination. Initiation is carried out by a fatty acid to amino acid coupling device that is comprised of a FAAL, a free-standing ACP, and a specialized C^III^ domain (FAAL:ACP:^III^C). Elongation is carried out by NRPS multi-modular proteins that utilize mostly CAT and CATE modules to insert specific l- and d-amino acids specified by A domain amino acid binding pocket codes. Termination is carried out by dimodular CAT-CATTe NRPS elongation proteins that have terminal Te domains for cyclization and release of finished lipopeptides. This lipopeptide assembly device is well suited for natural combinatorial evolution and combinatorial biosynthesis in the laboratory (Baltz, 2014b). The initiation and termination mechanisms have provided molecular beacons (FAAL, ACP, and CAT-CATTe proteins) for BLASTp searches of finished and draft genomes to identify cryptic known, related, and novel lipopeptide BGCs. This has been exemplified by identifying cryptic genes for several known BGCs (e.g., A54145, taromycin, laspartomycin/glycinocins, and telomycin), predicted BGCs (parvuline and amphomycin), a related BGC (daptomycin family), and two novel BGCs. Several of these were from draft genomes, and the large elongation NRPSs were fragmented, as anticipated (Klassen & Currie, 2012; Baltz, 2017b, 2018, 2019; Goldstein et al., 2019). As such, this work has identified previously unsequenced BGCs for known and novel lipopeptides that are candidates for finished genome sequencing and deposition in MIBiG to facilitate future comparative analyses (Kautsar et al., 2020). The new daptomycin-related BGC encoded by *S. sedi*, and novel BGCs encoded by *S. ambofaciens* and *S. zhaozhoensis* are candidates for fermentation/expression studies to identify new lipopeptides for biological testing.

It is noteworthy that all of the lipopeptide BGCs in this study appear to use either of two phylogenetically related ABC transporters for export and molecular target-agnostic resistance. No other potential resistance mechanisms are encoded in any of the BGCs. This is advantageous for ongoing natural evolution and laboratory-based combinatorial biosynthesis of new lipopeptides with new or improved biological activities, including possible new target interactions and MOAs, as anticipated from the proven diversity in MOAs within the group (Baltz, 2009; Johnston et al., 2016; Hover et al., 2018).

## Supplementary Material

kuab020_Supplemental_Files
