# Supplementary material for: Genome mining for drug discovery: cyclic lipopeptides related to daptomycin
Source: J Ind Microbiol Biotechnol. 2021 Mar 19;48(3-4):kuab020. doi: 10.1093/jimb/kuab020 (PMC9113097; doi:10.1093/jimb/kuab020)
Supplement: kuab020_Supplemental_Files [file kuab020_Supplemental_Files.zip › Supplemental Materials 7-24-2020.docx]

**Genome mining for drug discovery: cyclic lipopeptides related to daptomycin**

**Richard H. Baltz**

**CognoGen Biotechnology Consulting**

**7757 Uliva Way**

**Sarasota, FL 34238**

**E-mail: rbaltz923@gmail.com**

**Tel.: + 1-317-656-7601**

**Supplemental Materials**
