# Supplementary material for: Genome mining for drug discovery: cyclic lipopeptides related to daptomycin
Source: J Ind Microbiol Biotechnol. 2021 Mar 19;48(3-4):kuab020. doi: 10.1093/jimb/kuab020 (PMC9113097; doi:10.1093/jimb/kuab020)
Supplement: kuab020_Supplemental_Files [file kuab020_Supplemental_Files.zip › Table S1 Mol beacons lipopeptide BGCs 7-16-20.docx]

**Table S1** Molecular beacons for NRPS-derived peptide and lipopeptide BGCs

| Molecular beacon | BGC origin | Protein | Biosynthetic function/product | Target molecules | Ref |
| --- | --- | --- | --- | --- | --- |
| DptE  DptF  ACP-MP  DptD  LptD  PstD  PstA  TTe-MP  DptG  MbtH-MP  DptI  LptI  GlmT  DptJ  LptJ  LptK  LptL  GlmB  DptM  DptN  CAB38595  CAB38594  DptP  Tar5  Tar6  LipB | Daptomycin  Daptomycin  Multiple  Daptomycin  A54145  Friulimicin  Friulimicin  Multiple  Daptomycin  Multiple  Daptomycin  A54145  CDA  Daptomycin  A54145  A54145  A54145  Friulimicin  Daptomycin  Daptomycin  CDA  CDA  Daptomycin  Taromycin  Taromycin  Friulimicin | FAAL  ACP  ACP concatenate  NRPS  NRPS  NRPS  NRPS  PCP-Te concatenate  MbtH homolog  MbtH concatenate  α-KG methyltransferase  α-KG methyltransferase  α-KG methyltransferase  Trp 2,3-dioxygenase  L-Asp hydroxylase  L-hAsp methyltransferase  L-Asn hydroxylase  Glu mutase subunit  ABC transporter  ABC transporter  ABC transporter  ABC transporter  DedA superfamily  ACAD superfamily  ACAD superfamily  ACAD superfamily | FA-AA coupling  FA-AA coupling  FA-AA coupling  CAT-CATTe  CAT-CATTe  CAT-CATTe  ATTe  Peptide release  NRPS chaperone  NRPS chaperones  L-3mGlu_12_  L-3mGlu_12_  L-3mGlu_10_  L-Kyn_13_  L-hAsp_9_  L-moAsp_9_  L-hAsn_3_  L-3mAsp_4_  ATP-binding  Permease  ATP-binding  Permease  Export/resistance?  FA desaturation  FA desaturation  FA desaturation | Dpt-like, LPs  Dpt-like, LPs  LP families  Dpt-like, cLPs  Lpt-like, cLPs  Fri-like, cLPs  Fri-like  NRPs  NRPs  NRPs  Dpt-like  Lpt-like  CDA-like  Dpt-like  Lpt-like  Lpt-like  Lpt-like  Fri-like  Dpt-like, cLPs  Dpt-like, cLPS  CDA  CDA  Lpt, Dpt  LPs  LPs  LPs | 9  9  This report  9, 20  20  20  72, This report  18  11  12  9  20  20  9  20  20  20  20, 42  9  9  45, This report  45, This report  9  9, 90, This report  9, 90, This report  43, 72, This report |
