# Supplementary material for: Genome mining for drug discovery: cyclic lipopeptides related to daptomycin
Source: J Ind Microbiol Biotechnol. 2021 Mar 19;48(3-4):kuab020. doi: 10.1093/jimb/kuab020 (PMC9113097; doi:10.1093/jimb/kuab020)
Supplement: kuab020_Supplemental_Files [file kuab020_Supplemental_Files.zip › Table S2 DptF (ACP) multiprobe 3-24-20.docx]

**Table S2** DptF (ACP) homolog multiprobe (19-mer)

MNPPEAVSTPSEVTAWITGQIAEFVNETPDRIAGDAPLTDHGLDSVSGVALCAQVEDRYGIEVDPELLWSVPTLNEFVQALMPQLADRTMTPGVGENGSSERVGTWLVQQVSGFSGVAVDEIDVAAPLSEYGLNSVSALAICAAIEDEYEIEIEPTLLWDVESVSALTDAVVARLSMTMPDAGEKGSSDQIEKWLVQQVSAFSGVAAEQIDVTAPLSEYGLDSVSALAICAAIEDEYDIEIEPTLLWDVDTVSALVDSIVERLPASQDPEALRQRLRELCADCLGVPVDSLATDAPLTDYGMTSVTGTALCGMVEEYLDVECDLELLWQEPTIDGLASRLASRTVRMSLSPPSSPPPSPLAPGDPDALRQWLREQCADCLGVPAASLATDVPLTDYGMTSVTGTALCGVVEEYLGVECDLGLLWQEPTIDGLTVRLASRTVRMSLSPPPSPPAPRDPDALRQWLREQCADCLGVPAASLATDVPLTDYGMTSVTGTALCGMVEEHLAVECDLGLLWQEPTIDGLTARLAPRTVRMSPSPSPDHLRQWLREQCADCLGVPPESLATDIPLTDYGMTSVTGTALCGMVEDHLDVECDLGLLWQEQTIDAITSRLASRAERMSPSPSPSPEHLRQWLREQCADCLGVPPESLATDIPLTDYGMTSVTGTALCGMVEDHLDVECDLGLLWQEQTIDAITSRLASRAARMSDLSTAPTLDSLRVWLVDCVAGHLGLDAATIATDLPLTSYGLDSVYALSIAAELEDHLDVSLDPTLIWDHPTIDALSTALVAELRSAMSDLSTAPTLDDLRGWLIDCVAGHLGRDAATIATDVPLTSYGLDSVYALSIAAELEDHLDVSLDPTLIWDHPTIDALSTALMAELRSAMSLPDPGIETHTVEGLRAWLTDCVATHLDRPADTIDTSVRLTDYGLDSLYVLAVAAELEDHLDISLDPTLMWDNPTIDALSEALVAELAQTHAMSDPHAEPHTVDSLREWLTNCVASHLERSADTIDTSVRLSDYGLDSLYVLSVAGELEDHLDISLDPTLLWDNPTIDALSEALAQELAQYAMTDIGTDTGTHTVESLRSWLVDCVAAHLERPADTVDPAAKLSDYGLDSLYVLSVAAELEDHLDISLDPTVMWDNPTIDALSAALIRELAQQDMPETSTETIDVTALRNWLAGRIAEFTERPLAEIAGDKPLGEYGVDSVSALTVCAEIEDHFDITVEPTLLWDHPTIDAIAEVLVEEVNARMSQTSTQPIDVTTLRDWLTGRIAEFTERPPAEIAADKPLGEYGVDSVSALTVCAEIEDHFDITVEPTLLWDHPTIDAIAEALVDEVNARMTAHAHTVSEGAVRHWLAERLGVLLDRAGHEIQPDVLMAEYGLDSLQAVALAGEAEDRWGLPVDPDVAWEYPTVALLAAHLAARMADGGMTAHAHTVSERAVRHWLAERLGVLLDRAGHEIQPDVLMAEYGLDSLQAVALAGEAEDRWGLPVDPDVAWEYPTVALLAAHLAARMADGGMTAHSHTVSEIVVRHWLAERIGILLDRAGHEIQPDVLMAEYGLDSLQAVALAGEAEDRWGLPVDPDVAWQYPTVALLAAHLAARIADGDMSTHGASETEIRQWLTERIGDLIGDNAQEVLPDVLLAEYGVDSLQAFSLIGEIEDRWGLSLDAALTWEYPTIALLAAFLATEIAVDAVG
