# Supplementary material for: Genome mining for drug discovery: cyclic lipopeptides related to daptomycin
Source: J Ind Microbiol Biotechnol. 2021 Mar 19;48(3-4):kuab020. doi: 10.1093/jimb/kuab020 (PMC9113097; doi:10.1093/jimb/kuab020)
Supplement: kuab020_Supplemental_Files [file kuab020_Supplemental_Files.zip › Table S3 DptG 7-16-20.docx]

**Table S3** DptG (MbtH) homolog BLASTp scores for actinomycetes and uncultured bacteria

| Actinomycete | DptG homolog (predicted protein) | Query protein^b^ | | | | | | | |
| --- | --- | --- | --- | --- | --- | --- | --- | --- | --- |
|  |  | DptG | Tar11 | LptG | OrfX | Orf21 | SvH1 | Tem24 | MlcV |
| *S. roseosporus* NRRL 11379  *Sa. Sp.* CNQ490  *Sa. viridis* DSM 43017  *S. fradiae* A54145  *S. exfoliates* SM41693  *S. griseoluteus* ISP-5360  *S. pini* PL19  *S. barkulensis* RC 1830  *S. coelicolor* A3(2)  *S. lividans* TK24  *S.* sp. MBT28  *S.* sp. NRRL WC-3795  *A. friuliensis* DSM 7358  *UncBac* GQ475284  *S. viridochromogenes* ATCC 29814  *S. malaysiensis* DSM 4137  *S. sp.* M56  *S. sp.* SPMA113  *S. sp.* 1331.2  *S. canus* ATCC 12646  *S. canus* ATCC 12647  *S. qaidamensis* S10  *S. formicae* KY5  *UncBac* KY654519  *UncBac* KF264538  *S. fungicidicus* ATCC 21013  *S. canus* ATCC 12237  *S. parvulus* 2297  *S. ambofaciens* ATCC 23877  *S. zhaozhouensis* CGMCC 4.7095  *S. sedi* JCM 16909 | DptG  Tar11  (Tar11)  LptG  (LptG)  (LptG)  (LptG)  (LptG)  OrfX  OrfX  (OrfX)  (OrfX)  Orf21  Orf21  SvH1  (SvH1)  (SvH1)  (SvH1)  (SvH1)  Tem24  Tlo24  (Tem24)  (Tem24)  MlcV  MlcV  ABD65966 (ORF46)  KUN61419^a^  WP_114529433^a^  AKZ58684  WP_097229448  WP_139647435 | **100**  70  70  59  56  56  61  66  69  69  69  69  62  66  62  70  70  70  69  69  69  70  63  67  68  73  60  62  69  61  70 | 70  **100**  **90**  75  76  79  75  75  72  72  69  69  69  70  68  68  67  65  68  71  71  72  71  71  74  71  64  67  76  63  65 | 59  75  71  **100**  **91**  **91**  **81**  **84**  61  61  63  59  65  62  60  63  63  63  62  61  61  63  63  71  71  66  59  60  67  55  55 | 69  72  66  61  66  66  65  68  **100**  **100**  **96**  **96**  70  71  67  71  72  71  77  77  77  76  73  74  76  72  74  73  69  70  75 | 62  69  65  65  67  67  68  70  70  70  66  66  **100**  **93**  79  83  83  83  70  75  75  77  77  71  71  67  83  83  70  71  61 | 62  68  65  60  60  60  65  67  67  67  63  63  79  79  **100**  **84**  **84**  **84**  **80**  76  76  74  74  72  72  67  82  81  66  71  64 | 69  71  66  61  61  64  68  68  77  77  74  74  74  75  76  81  77  82  85  **100**  **99**  **97**  **85**  79  76  70  83  76  77  74  69 | 67  71  68  71  71  69  71  73  74  74  73  73  71  74  72  79  74  80  84  79  79  81  79  **100**  **96**  74  71  71  77  71  67 |

^b^ Possible orthologs are shown in **bold**

^a^ These proteins share 90% sequence identities
