# Supplementary material for: Genome mining for drug discovery: cyclic lipopeptides related to daptomycin
Source: J Ind Microbiol Biotechnol. 2021 Mar 19;48(3-4):kuab020. doi: 10.1093/jimb/kuab020 (PMC9113097; doi:10.1093/jimb/kuab020)
Supplement: kuab020_Supplemental_Files [file kuab020_Supplemental_Files.zip › Table S4 MbtH codes for lipopeptides 7-16-20.docx]

**Table S4** MbtH multiprobe codes for lipopeptides produced by actinomycetes or uncultured bacteria

| Actinomycete or uncultured bacterium | Lipopeptide | MbtH homolog | MbtH code^b^ |
| --- | --- | --- | --- |
| **Known and predicted producers**  *S. roseosporus* NRRL 11379  *S.* sp. CNQ490  *S. viridis* ATCC 43017  *S. fradiae* A54145  *S. exfoliatus* SM41693  *S. griseoluteus* ISP-5360  *S. pini* PL19  *S. barkulensis* RC 1830  *S. coelicolor* A3(2)  *S.* sp. MBT28  *A. friuliensis* DSM 7358  *UncBac* GQ475284  *S. viridochromogenes* ATCC 29814  *S. malaysiensis* DSM4137  *S.* sp. 1331.2  *UncBac* KY654519  *UncBac* KF264539  *S. canus* ATCC 12646  *S. canus* ATCC 12647  *S. qaidamensis* S10  *S. formicae* KY5  *S. fungicidicus*  **Consensus code**  **Other strains**  *S. canus* ATCC 12237  *S. parvulus* 2297  *S. ambofaciens* ATCC 23877  *S. zhaozhouensis* CGMCC 4.7095^a^  *S. sedi* JCM 16909 | Daptomycin  Taromycin  (desCl-Taromycin)  A54145  (A54145)  (A54145)  (A54145)  (A54145)  CDA  (CDA)  Friulimicin  Friulimicin  Laspartomycin  (Laspartomycin)  (Laspartomycin)  Malacidin  (Malacidin)  Telomycin  Telomycin  (Telomycin)  (Telomycin)  Enduracidin  Amphomycin  Parvuline?  Lipotridecapeptide  Unknown  Unknown | AAX31560  WP_024877506  WP_037312028  AAZ23079  WP_030556234  WP_030219293  WP_093851398  WP_101258560  AAD18046  WP_019328564  WP_023362362  ADK54912  AEG64691  WP_069871580  WP_09723931.1  ARU08091  AGS49343  AKQ13299  WP_059302640  WP_062930006  WP_09824074  ABD65966  KUN61419  WP_114529433  AKZ58684  WP_097229448  WP_139647435 | 332-333-322-333-322-222-223-3**2**2  332-333-322-333-322-222-223-312  332-333-322-333-322-222-223-3**2**2  332-333-**2**22-333-322-222-223-312  332-333-**2**22-333-322-222-223-312  332-333-**2**22-333-322-222-223-312  332-333-**2**22-333-322-222-223-312  332-333-322-333-322-222-223-312  332-333-322-333-322-222-223-312  332-333-322-333-322-222-223-312  3**2**2-3**2**3-322-333-322-222-223-312  3**2**2-3**2**3-322-333-322-222-223-312  3**2**2-3**2**3-322-333-32**1**-222-223-31**1**  332-333-322-333-322-222-223-312  3**2**2-**22**3-322-333-322-222-222-312  332-333-322-333-322-222-223-31**1**  332-333-32**1**-333-322-222-223-31**1**  332-333-322-333-322-222-223-312  332-333-322-333-32**1**-222-223-312  332-333-322-333-32**1**-222-223-312  332-333-322-333-322-222-223-312  332-333-**2**22-333-322-222-223-312  332-333-322-333-322-222-223-312  332-333-322-333-3**3**2-222-223-312  332-333-322-333-3**3**2-222-223-312  33**3**-333-322-333-322-222-223-312  332-333-3**1**2-333-32**1**-222-223-312  33**3**-333-322-333-322-222-223-312 |

^a^ *S. zhaozhouensis* encodes five MbtH homologs, each with a distinct MbtH code.

^b^ Deviations from the consensus lipopeptide MbtH code are shown in **bold.**
