# Supplementary material for: Genome mining for drug discovery: cyclic lipopeptides related to daptomycin
Source: J Ind Microbiol Biotechnol. 2021 Mar 19;48(3-4):kuab020. doi: 10.1093/jimb/kuab020 (PMC9113097; doi:10.1093/jimb/kuab020)
Supplement: kuab020_Supplemental_Files [file kuab020_Supplemental_Files.zip › Table S5 MbtH codes NRPS BGCs 7-16-20.docx]

**Table S5** MbtH consensus codes for diverse NRPS BGC families

| BGC family (numbers evaluated) | BGC type | MbtH consensus code^a^ | Divergence within family (%) | Divergence from lipopeptide family (%) |
| --- | --- | --- | --- | --- |
| Lipopeptide (21)  Vancomycin (5)  Bleomycin (6)  Griseobactin (5)  Nikomycin (2)  Nocardicin (2)  Pacidamycin (4) | Cyclic lipopeptide  Glycopeptide  PKS-glycopeptide  Catechol-peptide  Peptidyl-nucleoside  Beta-lactam  Uridylpeptide | 332-333-322-333-322-222-223-312  333-333-322-333-322-222-223-322  222-222-223-222-222-002-112-223  222-222-200-222-200-331-112-200  222-222-101-222-211-112-322-201  222-222-222-222-222-113-212-222  222-222-222-222-233-002-112-222 | 5.4  8.3  6.9  1.7  0.0  0.0  1.0 | -  8.3  83.3  95.8  87.5  70.8  79.2 |

^a^ MbtH multiprobe codes from Baltz [12]
