# Supplementary material for: Genome mining for drug discovery: cyclic lipopeptides related to daptomycin
Source: J Ind Microbiol Biotechnol. 2021 Mar 19;48(3-4):kuab020. doi: 10.1093/jimb/kuab020 (PMC9113097; doi:10.1093/jimb/kuab020)
Supplement: kuab020_Supplemental_Files [file kuab020_Supplemental_Files.zip › Table S6 DptM homolog ABC transporter 7-16-2020.docx]

**Table S6** DptM ABC transporter ATP-binding cassette homolog BLASTp scores in actinomycetes and uncultured bacteria

| Actinomycete or uncultured bacterium | DptM homolog (predicted protein) | Query protein^b^ | | | | | | | |
| --- | --- | --- | --- | --- | --- | --- | --- | --- | --- |
|  |  | DptM | Tar3 | LptM | CAB38595 | ExpB | Orf14 | Tem31 | MlcO |
| *S. roseosporus* NRRL 11379  *Sa. Sp.* CNQ490  *Sa. viridis* DSM 43017  *S. fradiae* A54145  *S. exfoliates* SM41693  *S. griseoluteus* ISP-5360  *S. pini* PL19139649596  *S. barkulensis* RC 1830  *S. coelicolor* A3(2)  *S.* sp. MBT28  *A. friuliensis* DSM 7358  *UncBac* GQ475284  *S. viridochromogenes* ATCC 29814  *S. malaysiensis* DSM 4137  *S. sp.* M56  *S. sp.* 1331.2  *S. canus* ATCC 12646  *S. canus* ATCC 12647  *S. qaidamensis* S10  *S. formicae* KY5  *UncBac* KY654519  *UncBac* KF264538  *S. canus* ATCC 12237  *S. parvulus* 2297  *S. ambofaciens* ATCC 23877  *S. zhaozhoouensis* CGMCC 4.7095  *S. sedi* JCM 16909 | DptM  Tar3  (Tar3)  LptM  (LptM)  (LptM)  (LptM)  (LptM)  CAB38595  CAB38595 ortholog  ExpB  ExpB  Orf14  (Orf14 ortholog)  (Orf14 ortholog)  (Orf14 ortholog  Tem31  Tlo31  (Tem31)  (Tem31)  MlcO  MlcO  (KUN68839)^a^  (WP_11453110)^a^  (AKZ58583)  (WP_097232900  (WP_13969596) | **100**  61  60  55  56  58  57  58  29  29  52  51  57  56  56  56  32  31  32  32  60  59  55  55  61  33  65 | 61  **100**  **85**  58  59  59  58  59  34  33  52  52  55  58  57  56  31  32  31  31  56  56  56  57  59  37  59 | 55  58  60  **100**  **88**  **89**  **73**  **79**  31  31  52  52  51  52  52  57  33  33  33  32  54  54  50  51  57  33  56 | 29  34  31  31  30  31  31  31  **100**  **96**  33  34  33  31  31  34  65  65  66  66  29  29  33  33  30  69  29 | 52  54  52  52  51  53  51  52  33  29  **100**  **95**  58  60  60  61  29  29  29  32  57  56  58  59  52  32  53 | 57  55  56  52  54  53  55  55  34  31  58  57  **100**  **84**  **84**  **80**  33  34  33  31  59  59  73  74  55  36  58 | 31  31  32  33  35  35  34  34  65  65  29  33  33  33  33  35  **100**  **99**  **95**  **84**  31  31  32  22  32  61  31 | 60  56  58  54  54  55  57  57  29  29  57  56  59  57  57  60  31  31  32  31  **100**  **93**  56  57  62  34  61 |

^a^ These proteins share 89% sequence identities

^b^ Possible orthologs are shown in **bold**
