# Supplementary material for: Genome mining for drug discovery: cyclic lipopeptides related to daptomycin
Source: J Ind Microbiol Biotechnol. 2021 Mar 19;48(3-4):kuab020. doi: 10.1093/jimb/kuab020 (PMC9113097; doi:10.1093/jimb/kuab020)
Supplement: kuab020_Supplemental_Files [file kuab020_Supplemental_Files.zip › Table S7 DptN ABC transporter homologs 7-16-20.docx]

**Table S7** DptN ABC transporter permease homolog BLASTp scores in actinomycetes and uncultured bacteria

| Actinomycete or uncultured bacterium | DptN homolog (predicted product) | Query protein^b^ | | | | | | | |
| --- | --- | --- | --- | --- | --- | --- | --- | --- | --- |
|  |  | DptN | Tar2 | LptN | CAB-  38594 | ExpA | Orf20 | Tem30 | MlcD |
| *S. roseosporus* NRRL 11379  *Sa. Sp.* CNQ490  *Sa. viridis* DSM 43017  *S. fradiae* A54145  *S. exfoliates* SM41693  *S. griseoluteus* ISP-5360  *S. pini* PL19  *S. barkulensis* RC 1830  *S. coelicolor* A3(2)  *S.* sp. MBT28  *A. friuliensis* DSM 7358  *UncBac* GQ475284  *S. viridochromogenes* ATCC 29814  *S. malaysiensis* DSM 4137  *S. sp.* M56  *S. sp.* 1331.2  *S. canus* ATCC 12646  *S. canus* ATCC 12647  *S. qaidamensis* S10  *S. formicae* KY5  *UncBac* KY654519  *UncBac* KF264538  *S. canus* ATCC 12237  *S. parvulus* 2297  *S. ambofaciens* ATCC 23877  *S. zhaozhouensis* CGNCC 4.7095  *S. sedi* JCM 16909 | DptN  Tar2  (Tar2)  LptN  (LptN)  (LptN)  (LptN)  (LptN)  CAB38594  CAB38594 ortholog  ExpB  ExpB  Orf14  (Orf14 ortholog)  (Orf14 ortholog)  (Orf14 ortholog  Tem30  Tlo30  (Tem30)  (Tem30)  MlcO  MlcO  KUN68834^a^  WP_114531154^a^  AKZ58682  WP_07232901  WP_139649594 | **100**  65  62  56  59  59  60  56  -  -  50  50  47  50  50  56  -  -  -  -  56  54  50  50  63  -  **70** | 62  **100**  **86**  54  58  58  59  62  -  -  54  54  48  50  50  54  -  -  -  -  50  50  51  50  61  -  57 | 59  58  62  **100**  **91**  **92**  **90**  **85**  -  -  51  52  47  51  51  54  29  29  31  38  51  53  51  50  59  -  60 | **-**  **-**  **-**  **-**  **-**  **-**  **-**  **-**  **100**  **92**  -  -  -  -  -  -  42  42  41  42  -  -  **-**  **-**  **-**  58  **-** | 50  54  53  51  52  53  54  56  **-**  **-**  **100**  **95**  68  67  67  **75**  -  -  -  -  48  48  71  70  52  -  52 | 48  47  47  47  48  48  50  49  -  -  68  68  **100**  **82**  **82**  **77**  **-**  **-**  **-**  **-**  47  47  73  73  52  -  47 | -  -  -  -  -  -  -  -  42  42  -  -  -  -  -  -  **100**  **99**  **94**  **75**  **-**  **-**  **-**  **-**  **-**  38  **-** | 57  50  50  51  52  52  50  49  -  -  49  49  47  49  49  51  **-**  **-**  **-**  **-**  **100**  **96**  49  48  56  -  55 |

^a^ These proteins share 89% sequence identities

^b^ Possible orthologs are shown in bold
