# Supplementary material for: Genome mining for drug discovery: cyclic lipopeptides related to daptomycin
Source: J Ind Microbiol Biotechnol. 2021 Mar 19;48(3-4):kuab020. doi: 10.1093/jimb/kuab020 (PMC9113097; doi:10.1093/jimb/kuab020)
Supplement: kuab020_Supplemental_Files [file kuab020_Supplemental_Files.zip › Table S8 DptP transport-resistance 7-16-20.docx]

**Table S8** DptP homolog BLASTp scores for actinomycetes

| Actinomycete | DptP homolog (predicted) | Query protein^a^ | | | |
| --- | --- | --- | --- | --- | --- |
|  |  | DptP | Tar1 | (Tar1) | LptP |
| *S. roseosporus* NRRL 11379  *Sa. Sp.* CNQ490  *Sa. viridis* DSM 43017  *S. fradiae* A54145  *S. exfoliates* SM41693  *S. griseoluteus* ISP-5360  *S. pini* PL19  *S. barkulensis* RC 1830  *S. sedi* JMC 16909 | DptP  Tar1  (Tar1)  LptP  (LptP)  (LptP)  (LptP)  (LptP)  - | **100**  47  49  **94**  **91**  **92**  **81**  **81**  - | 47  **100**  **79**  55  56  52  52  53  - | 49  **79**  **100**  55  59  53  52  53  - | **94**  51  50  **100**  **91**  **92**  **80**  **80**  **-** |

^a^ Possible orthologs are in **bold**
