# Supplementary material for: Genome mining for drug discovery: cyclic lipopeptides related to daptomycin
Source: J Ind Microbiol Biotechnol. 2021 Mar 19;48(3-4):kuab020. doi: 10.1093/jimb/kuab020 (PMC9113097; doi:10.1093/jimb/kuab020)
Supplement: kuab020_Supplemental_Files [file kuab020_Supplemental_Files.zip › Table S9 PstA homologs 7-16-20.docx]

**Table S9** NRPS PstA BLASTp scores in actinomycetes and uncultured bacteria

| Actinomycete | PstA homolog (predicted)^a^ | Query protein^c^ | | | | | |
| --- | --- | --- | --- | --- | --- | --- | --- |
|  |  | PstA | LpmA | “GlyA” | (“AmpA”) | (“ParA”) | MlcA |
| *A. friuliensis* DSM 7358  *UncBac* GQ475284  *S. viridochromogenes* ATCC 29814  *S.* sp. M56  *S. malaysiensis* DSM 4137  *S. canus* ATTC 12237  *S. parvulus* 2297  *UncBac* KY654519  *UncBac* KF264539 | PstA  PstA  LpmA^b^  (“GlyA”)  (“GlyA”)  (“AmpA”)  (“ParA”)  MlcA  MlcA | **100**  **94**  59  57  57  61  65  61  64 | 59  59  **100**  69  69  66  67  54  53 | 58  58  69  **100**  **99**  67  66  50  51 | 65  65  66  67  67  **100**  **84**  55  55 | 65  64  67  66  66  **84**  **100**  56  55 | 54  56  53  50  52  56  55  **100**  **91** |

^a^ PstA homologs for NRPS proteins (ATTe) predicted to be involved in biosynthesis of glycinocin, amphomycin, and parvuline are designated as “GlyA”, “AmpA”, and “ParA”. PstA homologs are not encoded by *S. ambofaciens* ATCC 23877, *S. zhaozhouensis* CGMCC 4.7095, or *S. sedi* JMC 16909.

^b^ LpmA (942 amino acids) is truncated by 120 amino acids at the C-terminus relative to PstA (1042 amino acids), and lacks the thioesterase domain Te of the ATTe. All others have complete ATTe structures.

^c^ Possible orthologs are in bold.
