# Supplementary material for: Genome mining for drug discovery: cyclic lipopeptides related to daptomycin
Source: J Ind Microbiol Biotechnol. 2021 Mar 19;48(3-4):kuab020. doi: 10.1093/jimb/kuab020 (PMC9113097; doi:10.1093/jimb/kuab020)
Supplement: kuab020_Supplemental_Files [file kuab020_Supplemental_Files.zip › Table S10 PstB homologs 7-16-20.docx]

**Table S10** NRPS PstB BLASTp scores in actinomycetes with finished genomes and uncultured bacteria

| Actinomycete | PstB homolog^a^ | Query prote | | | |
| --- | --- | --- | --- | --- | --- |
|  |  | PstB | LpmB | “GlyB”^b^ | MlcK |
| *Actinoplanes friuliensis*  *UncBac* GQ475284  *S. viridochromogenes* ATCC 29814  *S.* sp. M56  *S. malaysiensis* DSM 4137  *UncBac* KY654519  *UncBac*^c^ KF264539 | PstB  PstB  LpmB  (“GlyB”)  (“GlyB”)  MlcK  (MlcK) | **100**  **92**  61  64  64  49  49 | 61  61  **100**  **76**  **76**  50  50 | 64  64  **77**  **100**  **99**  51  51 | 49  49  50  51  51  **100**  **89** |

^a^ The PstB NRPS homolog (CAT-CT-CATTe) predicted to be involved in glycinocin biosynthesis is designated as “GlyB”.

^b^ Possible orthologs are in bold
